# Purinergic signaling modulates CD4^+^ T cells with cytotoxic potential during *Trypanosoma cruzi* infection

**DOI:** 10.1172/JCI186785

**Published:** 2025-07-01

**Authors:** Gastón Bergero, Yanina L. Mazzocco, Sebastian Del Rosso, Ruining Liu, Zoé M. Cejas Gallardo, Simon C. Robson, Martin Rottenberg, Maria P. Aoki

**Affiliations:** 1 Universidad Nacional de Córdoba, Facultad de Ciencias Químicas, Departamento de Bioquímica Clínica, Córdoba, Argentina.; 2 Consejo Nacional de Investigaciones Científicas y Técnicas (CONICET), Centro de Investigación en Bioquímica Clínica e Inmunología (CIBICI), Córdoba, Argentina.; 3 Department of Microbiology, Tumor and Cell Biology, Karolinska Institutet. Stockholm, Sweden.; 4 Center for Inflammation Research, Department of Anesthesia, Critical Care & Pain Medicine, Beth Israel Deaconess Medical Center, Harvard Medical School, Boston, Massachusetts, USA.

**Keywords:** Immunology, Infectious disease, Inflammation, Hypoxia, Parasitology, T cells

## Abstract

Chagas disease, caused by *Trypanosoma cruzi*, is endemic to Latin America and is characterized by chronic inflammation of cardiac tissues due to parasite persistence. Hypoxia within infected tissues may trigger the stabilization of HIF-1 and be linked to ATP release. Extracellular ATP exhibits microbicidal effects but is scavenged by CD39 and CD73 ectonucleotidases, which ultimately generate adenosine (ADO), a potent immunosuppressor. Here, we comprehensively study the importance of HIF-1 stabilization and the CD39/CD73/ADO axis, on CD4^+^ T cells with the cytotoxic phenotype, in facilitating the persistence of *T. cruzi*. Myocardial infection induces prominent areas of hypoxia, which is concomitant with HIF-1α stabilization in T cells and linked to early expansion of CD39^+^CD73^+^CD4^+^ T cell infiltrating population. Functional assays further demonstrate that HIF-1 stabilization and CD73 activity are associated with impaired CD4^+^ T cell cytotoxic potential. RNA-Seq analysis reveals that HIF-1 and purinergic signaling pathways are overrepresented in cardiac tissues of patients with end-stage Chagas disease. The findings highlight a major effect of purinergic signaling on CD4^+^ T cells with potential cytotoxic capacity in the setting of *T. cruzi* infection and have translational implications for therapy.

## Abstract 2

We found compelling evidence that hypoxia modulates the CD4 T-cells with cytotoxic potential by triggering, in a time-dependent manner, purinergic signaling during *Trypanosoma cruzi* infection.

## Graphical abstract

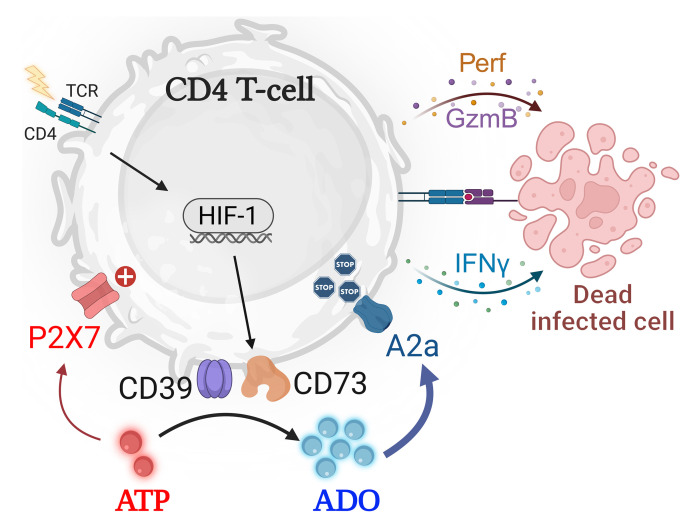

## Introduction

Chronic Chagas cardiomyopathy (CCC), caused by the intracellular protozoan parasite *Trypanosoma cruzi*, is the major cause of infectious myocarditis worldwide (1). Cell-mediated immunity, primarily orchestrated by CD4^+^ T cells and macrophages, controls parasite levels but cannot fully clear the infection. The inability to eradicate infection suggests an excessive regulation of immune effector mechanisms, ultimately leading to the progression of the disease into its chronic phase.

After infection, the influx of immune cells and altered blood flow result in local hypoxia, a stimulus that stabilizes the transcription factor hypoxia-inducible factor-1α (HIF-1α). Stabilized HIF-1α translocates to the nucleus and activates the expression of target genes (2). HIF-1 has been shown to enhance effector T cell function, promoting cytolytic activity, inflammatory cytokine production (3, 4), and T cell survival (5). These pathophysiological stress-adaptive responses are well recognized in tumors (6), but contributions to the immune response in determining the persistence or the clearance of microorganisms are not completely understood.

Hypoxic cells promptly respond to inflammatory environments by releasing ATP, leading to extracellular ATP (eATP) levels that typically reach high concentrations (hundred micromolar), in contrast to approximately 10 nM found in physiological conditions (7). eATP acts as a signaling molecule inducing proinflammatory and microbicidal effects acting on P2 receptors. Among them, P2X7 receptor (P2X7R) is particularly significant because of its role in inflammatory pathological conditions (8). Stimulation of P2X7R with low doses of ATP triggers the opening of a permeable channel to Na^+^, K^+^, and Ca^2+^ ions, but sustained stimulation with high ATP concentrations leads to the formation of a non-selective pore and extensive ATP release that further amplifies purinergic signaling (9). However, the half-life of ATP is extremely short, as it is hydrolyzed by the ecto-apyrase (CD39) and the ecto-5′-nucleotidase (CD73) enzymes, which convert ATP to adenosine (ADO) (10). Although ADO is well recognized as a molecular building block of the genetic code (11), there are 4 ADO receptor subtypes; among them, the A2a and A2b receptors (A2aR and A2bR) respond to limitation of inflammation (12). Hypoxia has been implicated in enhancing the enzymatic capacity of CD39 and CD73 (13, 14), mitigating excessive inflammation. In this context, eATP can activate HIF-1 (15), establishing a regulatory loop controlled by purinergic machinery. The coexpression of CD39 and CD73 ectonucleotidases is a hallmark of murine FoxP3^+^ Tregs (16). Although their expressions have also been reported in nonregulatory T cells (17, 18), the role of these enzymes in the function of effector T cells, as in *T. cruzi* infection, needs further detailed investigation.

CD4^+^ T cells perform an extensive variety of functions and are best known for their role as T helper (Th) cells through cytokine release. However, CD4^+^ T cells with cytotoxic activity (CD4 CTLs) characterized by the ability to secrete granzyme B, perforin, and IFN-γ and to kill target cells in an MHC class II–restricted fashion were observed in a range of immune responses (19) and expanded in supercentenarians (20), and their importance in Chagas disease has been recently revealed (21). Considering that CD4 CTLs’ differentiation mechanism remains elusive (22), it is critical to define the signals required for their development.

Purinergic signaling is involved in both the physiology and pathophysiology of the heart (23). We have previously reported that pharmacological inhibition of CD73 activity early after *T. cruzi* infection delayed the progression of experimental Chagas cardiomyopathy (24). Additionally, we have found that increased expression of HIF-1α and CD73 in infiltrating leukocytes, predominantly T cells, within cardiac explants from patients with CCC correlates with disease severity and local parasite load (25). These findings underscore the pivotal role of purinergic signals in regulating T cell functions and influencing disease outcomes and prompted us to go deeper into understanding the immune mechanisms underlying the development of Chagas disease.

In the present study, we have further investigated the influence of the purinergic system on the CD4^+^ T cells with potential cytotoxic capacity in the context of murine in vitro activation and in vivo infection with *T. cruzi*. Our findings validate dynamic changes of purinergic system components upon cellular activation. Infection with *T. cruzi* expands a CD39^+^CD73^–^P2X7^+^ CD4^+^ T cell population expressing the granzyme B effector molecule. Unexpectedly, we found that HIF-1 stabilization is associated with impaired cellular activation and proliferation, which is concurrent with the upregulation of CD73 expression. Strikingly, RNA-Seq analysis revealed that the HIF-1 and purinergic signaling pathways were among the most differentially expressed pathways in the cardiac tissues of CCC patients. Overall, our findings highlight the impact of purinergic signaling in driving CD4^+^ T cell effector and cytotoxic responses and provide insights into potential purinergic-targeting therapies in the setting of an intracellular parasitic infection.

## Results

### Dynamic purinergic signaling events during CD4^+^ T cell activation.

**Figure 1:**
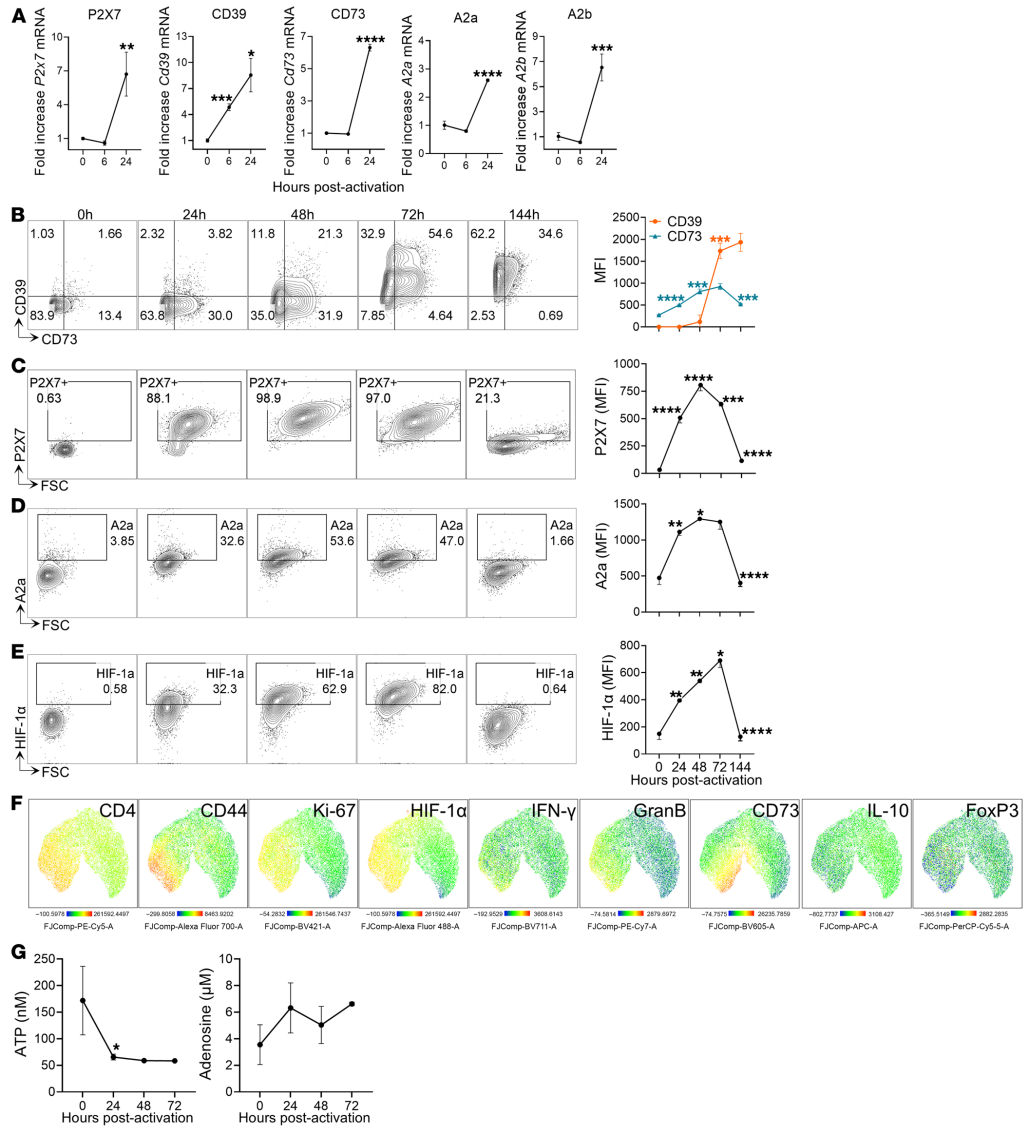
Dynamic of purinergic signaling components during CD4^+^ T cell activation. (**A**) Fold changes of mRNA expression of P2X7R, CD39, CD73, A2aR, and A2bR in splenic naive CD4^+^ T cells at 0, 6, and 24 hours after stimulation (*n* = 3 biological samples in 3 replicates). mRNA gene expression levels were normalized to that in naive cells. (**B**–**E**) Representative contour plots and MFIs of CD39 and CD73 (**B**), P2X7R (**C**), A2aR (**D**), and HIF-1α (**E**) expression from 0 to 144 hours after stimulation, measured by flow cytometry (*n* = 3–4 per time). (**F**) UMAP plots of FACS data of the total CD4^+^ T cells at 72 hours after stimulation. UMAPs are color-coded according to expression levels of the antigen listed at the top (red corresponding to the highest expression and blue the lowest). (**G**) ATP and ADO levels in the culture supernatant at indicated time points (*n* = 3–4 per time). Independent-samples *t* test was performed to compare each time point with the previous one (**A**–**G**). *P < 0.05, **P < 0.01, ***P < 0.001, ****P < 0.0001.

To gain insight into the dynamics of ATP metabolism during the activation of CD4^+^ T cells, we investigated the kinetic profiles of CD73 and CD39 ectoenzymes, along with the ATP receptor P2X7 and ADO receptors A2a and A2b, after anti-CD3 and -CD28 stimulation of splenic naive CD4^+^ T cells. We observed an increase in the mRNA levels of CD39 and CD73 ectoenzymes, P2X7R, and A2a and A2b receptors 24 hours after T cell receptor (TCR) activation, ranging from 2 to 7 times greater than those in non-stimulated CD4^+^ T cells (Figure 1A). Following the gating strategy shown in Supplemental Figure 1A (supplemental material available online with this article; https://doi.org/10.1172/JCI186785DS1), we determined that non-stimulated CD4^+^ T cells were predominantly CD39- and CD73-negative. Upon activation, CD39^+^CD73^+^ cells increased, peaking at 72 hours after stimulation and predominating over single-positive populations. Notably, CD73 expression increased after activation, peaked at 72 hours, and decreased at 144 hours (MFI = 809 ± 55 at 48 hours, 922 ± 66 at 72 hours, and 524 ± 16 at 144 hours). In contrast, CD39 expression showed a robust increase at 72 hours and remained high until 144 hours after activation (MFI = 116 ± 157 at 48 hours, 1,736 ± 175 at 72 hours, and 1,932 ± 204 at 144 hours) (Figure 1B and Supplemental Figure 1B).

In contrast to non-stimulated CD4^+^ T cells, which express extremely low levels of P2X7R and A2aR, the expression of these receptors significantly increased upon TCR stimulation. Importantly, 24 hours after activation most cells were P2X7^+^, which validates a key role for eATP signaling during T cell activation (Figure 1C). The expression levels of both receptors peaked at 48 hours, and declined thereafter, reaching similar levels to those in unstimulated cells at 144 hours (Figure 1, C and D, and Supplemental Figure 1, C and D). The changes in the expression of purinergic enzymes and receptors suggest a dynamic sensitivity of CD4^+^ T cells to the purinergic milieu during the activation process.

Considering that P2X7R activation by eATP can stimulate HIF-1 signaling (26), we analyzed the progression of the HIF-1α subunit during cell activation. Consistent with previous reports (27), CD4^+^ T cells rapidly increased HIF-1α levels at 24 hours, and this increase persisted until 72 hours after TCR stimulation (Figure 1E and Supplemental Figure 1E). High-dimensional and uniform manifold approximation and projection (UMAP) analysis revealed that HIF-1α was expressed in activated CD4^+^ T cells (CD44^+^), and that activated cells were proliferating (Ki-67^+^) and expressing effector molecules (granzyme B^+^ and IFN-γ^+^) and the CD73 ectoenzyme (Figure 1F). Considering that classical FoxP3^+^ regulatory T cells (Tregs) and FoxP3^–^IL-10^+^ type 1 regulatory (Tr1) cells express granzyme B, we analyzed these populations and found that they represented less than 5% of the total CD4^+^ T cells. Furthermore, most granzyme B^+^ cells were negative for FoxP3 and IL-10 expression (Figure 1F and Supplemental Figure 1, F and G), evidencing that conventional CD4^+^ T cells, but no regulatory ones, account for the majority of granzyme B production. At 144 hours, in line with CD73, P2X7R, and A2aR expression, HIF-1α expression drastically decreased (Figure 1E), evidencing a close interplay among these components.

Finally, we evaluated the extracellular levels of nucleotide and nucleoside metabolites during T cell activation. In agreement with the lower basal ectoenzyme expression, the highest levels of eATP (171 ± 64 nM) and the lowest levels of extracellular ADO (eADO) (3.5 ± 2.5 μM) were detected under such non-stimulatory conditions. However, after activation-induced ectoenzyme expression, eATP levels decreased to 58 ± 1 nM, while ADO increased to 6.6 ± 0.2 μM (Figure 1G), suggesting important impacts of differential levels of CD73 and CD39 expression during CD4^+^ T cell activation.

### Extracellular ATP and ADO differentially impact CD4^+^ T cell activation and proliferation.

**Figure 2:**
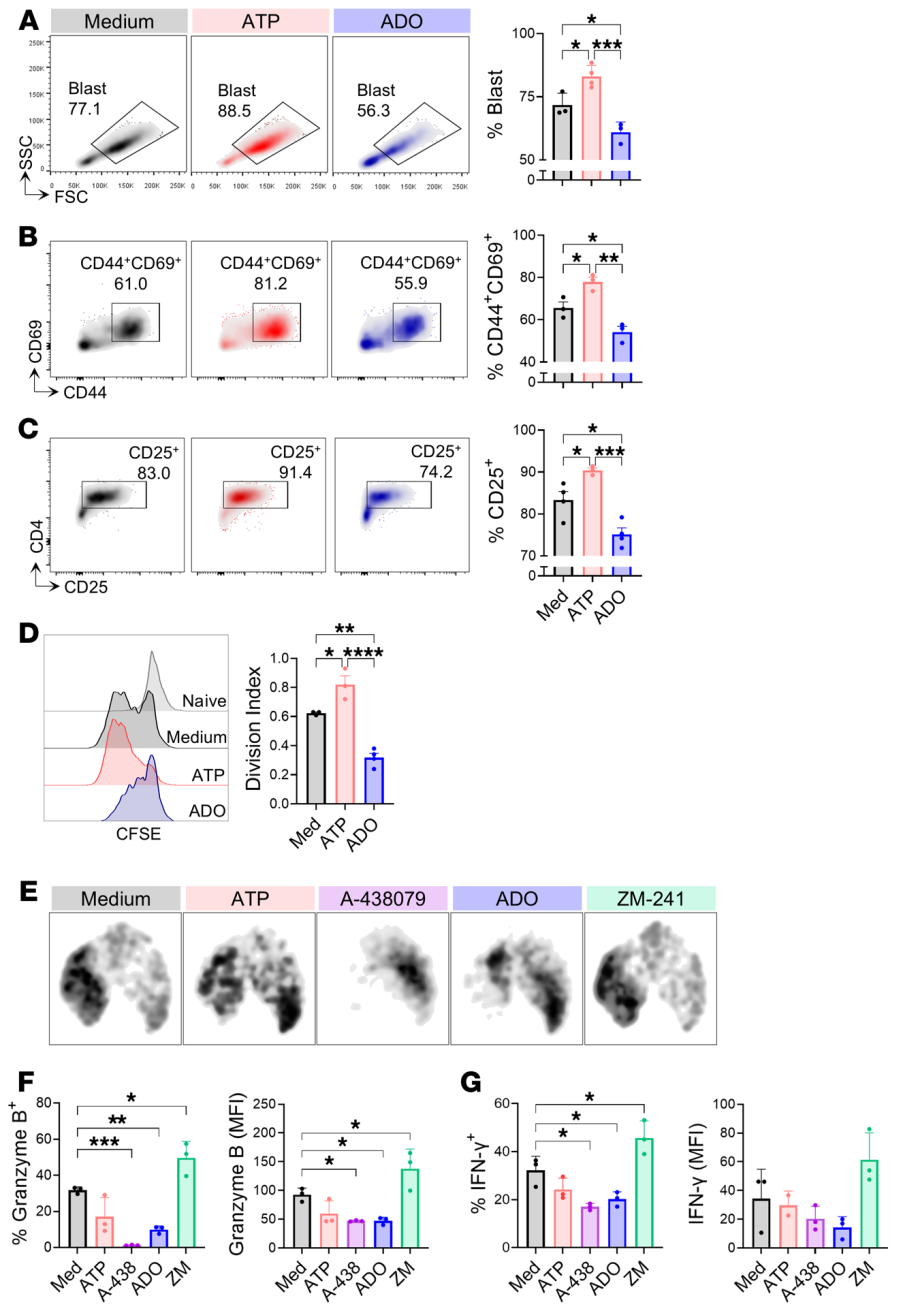
Extracellular ATP and ADO differentially impact CD4^+^ T cell activation and proliferation. (**A**–**D**) Naive CD4^+^ T cells were stimulated with anti-CD3/CD28 antibodies and supplemented with ATP (250 nM) or ADO (500 μM) for 72 hours (*n* = 4 per group). Representative dot plots and percentages of blasts (**A**), activation surface markers CD44^+^CD69^+^ (**B**), and CD25^+^ (**C**) in CD4^+^ T cells. (**D**) Representative histograms and quantification of the proliferation rate via CFSE dilution of CD4^+^ T cells. (**E**–**G**) CD4^+^ T cells were stimulated with anti-CD3/CD28 antibodies and supplemented with ATP, ADO, A2aR inhibitor ZM-241385 (1 μM), or P2X7R inhibitor A-438079 (25 μM) or maintained in medium alone for 72 hours (*n* = 3 per group). UMAP plots (**E**) and percentages and MFIs of granzyme B (**F**) and IFN-γ (**G**) expression in CD4^+^ T cells. One-way ANOVA followed by Tukey’s post hoc test was conducted for **A**–**G**. *P < 0.05, **P < 0.01, ***P < 0.001, ****P < 0.0001.

Considering that purine metabolism increases upon T cell activation and its direct impacts on the immune response, we investigated the biological effects of eATP and eADO signaling on CD4^+^ T cells. To test this, naive CD4^+^ T cells were TCR-stimulated for 72 hours in the presence of ATP or ADO, and their activation and proliferation rate were determined as shown in Supplemental Figure 2, A–D. In accordance with prior studies from Trabanelli and collaborators (28), we found that the effect of eATP on T cells was concentration dependent. ATP within the “physiological ranges” proposed for the extracellular milieu (50 nM) did not affect CD4^+^ T cell activation or proliferation, whereas at “pathological concentrations” (100 μM), ATP suppressed both endpoints (Supplemental Figure 2, E–G). Conversely, the addition of ATP at levels detected in an “inflammatory environment” as a result of a controlled release (29) (250 nM) increased the cell size (evaluated as the percentage of blasts), the surface expression of activation markers (CD44 and CD69), and the IL-2 receptor component CD25, as well as the proliferative capacity, in comparison with those cells only stimulated with anti-CD3/CD28 (Figure 2, A–D).

Next, we assessed the effect of ADO on CD4^+^ T cell responses to TCR stimulation. While addition of 100 μM ADO did not exhibit an inhibitory effect, 1 mM ADO completely suppressed the T cell activation (Supplemental Figure 2, H and I). The incubation with 500 μM ADO significantly reduced cell activation and proliferation (Figure 2, A–D).

To evaluate whether ATP and ADO signaling impacts T cell effector functions, purified CD4^+^ T cells were TCR-stimulated in the presence of ATP (250 nM), ADO (500 μM), the A2aR inhibitor ZM-241385 (ZM-241), or the P2X7R inhibitor A-438079 (A-438) for 72 hours. The UMAP analysis (Figure 2E) revealed that stimulated cells were primarily localized in the region associated with an activated phenotypic profile (see Figure 1F). The density of this region was markedly reduced by A-438 and ADO treatments and increased by ZM-241. Notably, while ATP cannot stimulate, the specific inhibition of P2X7R prevented the expression of effector molecules such as granzyme B and IFN-γ (Figure 2, F and G). The lack of effects of ATP incubation could indicate its metabolization toward the production of ADO. To test this hypothesis, and given that CD73 is the crucial enzyme responsible for generating eADO from eATP, we analyzed CD4^+^ T cells incubated with APCP (a specific CD73 inhibitor), or CD4^+^ T cells from *Cd73^–/–^* spleens. As expected, the pharmacological or genetic abolition of CD73 activity resulted in enhanced CD4^+^ T cell activation (data not shown). These data suggest that P2X7 receptor signaling is required for the efficient activation of T cells by eATP.

In contrast, ADO stimulation induced a significant diminution in the frequency of cells producing granzyme B and IFN-γ, an effect that was reversed by the incubation with the inhibitor ZM-241, indicating that ADO-mediated inhibition of CD4^+^ T cell activation is through the A2a receptor (Figure 2, F and G). In summary, these findings highlight the crucial role of the purinergic system in modulating the activation and proliferation of CD4^+^ T cells with cytotoxic potential.

### HIF-1α stabilization potentiates the effects of CD73.

**Figure 3:**
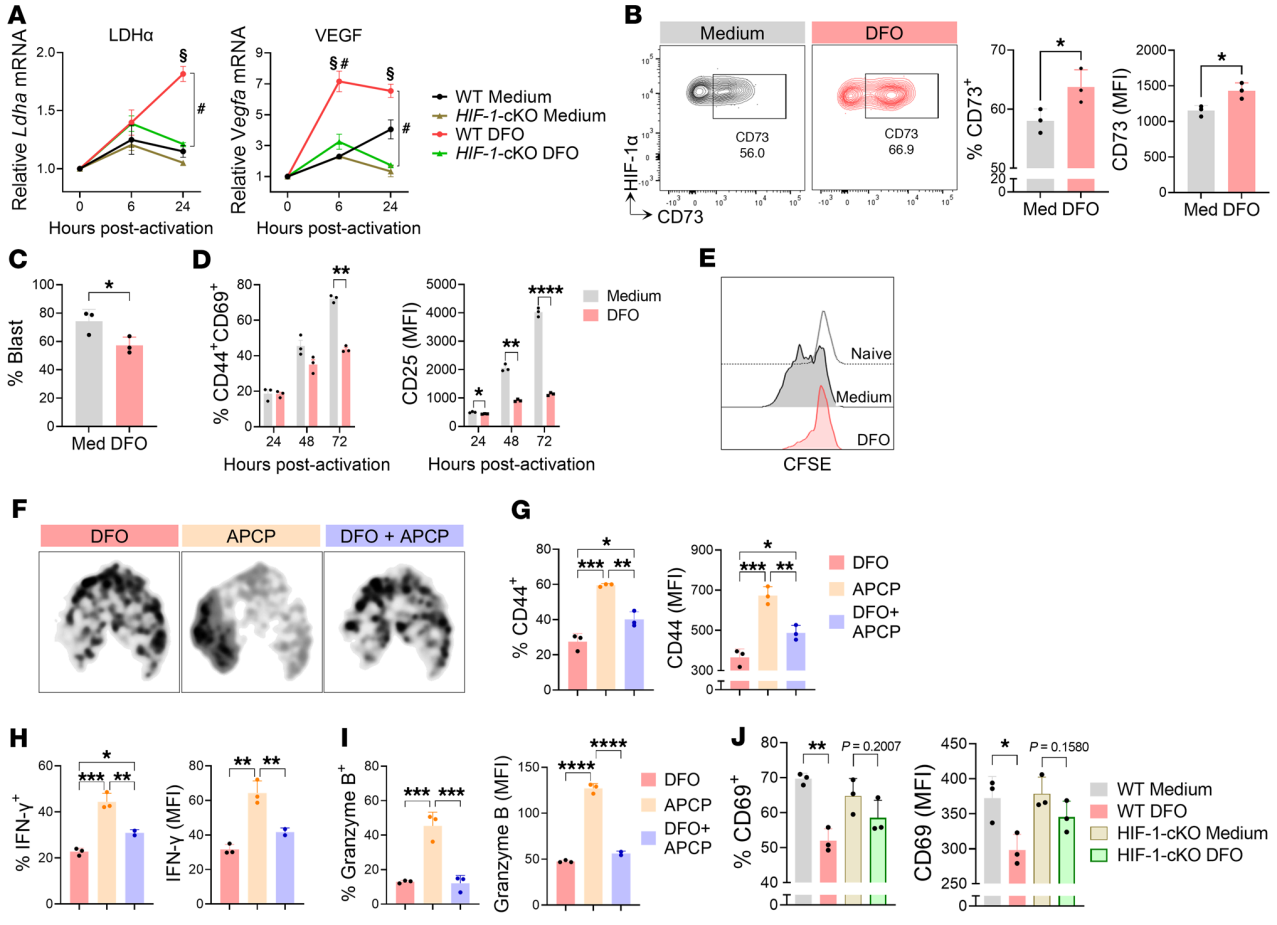
HIF-1α stabilization potentiates the effects of CD73. Naive CD4^+^ T cells were cultured with anti-CD3/CD28 in the presence of the HIF-1α stabilizer DFO (5 μM) or maintained in medium alone. (**A**) *Ldha* and *Vegf* mRNA levels in *Hif-1^+/+^* (WT) or *Hif-1^–/–^* (*HIF-1-cKO*) CD4^+^ T cells determined by real-time PCR and normalized to those in naive cells (*n* = 3 biological samples in 3 replicates). (**B**) Representative contour plots and quantification of CD73 expression in HIF-1α^+^CD44^+^CD4^+^ T cells at 72 hours after activation evaluated by flow cytometry. (**C**) Frequency of blast cells at 72 hours after activation. (**D**) Percentage of CD44^+^CD69^+^ cells and the MFI of CD25 at indicated time points. (**E**) Representative histograms of the proliferation rate via CFSE dilution at indicated conditions at 72 hours after activation. (**F**–**I**) CD4^+^ T cells were activated for 72 hours in the presence of DFO, the CD73 inhibitor APCP (100 μM), or both. UMAP analysis (**F**) and quantification of CD44 (**G**), IFN-γ (**H**), and granzyme B (**I**) expression. (**J**) CD69 expression in DFO-treated WT or *HIF-1-cKO* CD4^+^ T cells after 72 hours of activation. *n* = 3 in all panels. Independent-samples *t* tests were performed to compare WT-DFO vs. WT-medium (^§^*P* < 0.05) and WT-DFO vs. HIF-1-cKO-DFO (^#^*P* < 0.05) (**A**). Independent *t* test was performed to compare medium vs. DFO in **B**–**D** and **J**. One-way ANOVA followed by Tukey’s post hoc test was conducted for **G**–**I**. *P < 0.05, **P < 0.01, ***P < 0.001, ****P < 0.0001.

Considering the multiple roles of HIF-1 in immune response modulation (2), we investigated whether HIF-1 regulates the suppressive effects of eADO. We modeled the impacts of HIF-1 stabilization on CD4^+^ T cell function using the iron chelator deferoxamine (DFO), which stabilizes HIF by inhibiting prolyl hydroxylases in normoxic conditions (30). DFO-treated, TCR-stimulated CD4^+^ T cells exhibited increased expression of the HIF-1 target genes lactate dehydrogenase A (*Ldha*) and vascular endothelial growth factor (*VEGF*), compared with untreated cells (Figure 3A), confirming effective HIF-1 stabilization. Importantly, CD4^+^ T cells obtained from *Hif1a^fl/fl^* *Cd4^cre^* conditional mice (HIF-1–cKO) showed no increase of these genes in response to DFO, showing that HIF-1 mediated responses to DFO.

The treatment with DFO elevated CD73 protein expression 72 hours after activation (Figure 3B), coinciding with impaired activation and proliferation (Figure 3, C–E). According to the spatial segregation shown in UMAP plots (Figure 3F), subsequent CD73 inhibition in DFO-treated T cells partially restored activation and IFN-γ production, albeit without affecting granzyme B expression (Figure 3, G–I). Notably, compared with control cells, *HIF-1-cKO* cells treated with DFO exhibited comparable CD69 expression (Figure 3J). Overall, these findings suggest the active involvement of HIF-1 in modulating CD4^+^ T cells with cytotoxic potential through CD73 activity.

### Genetic HIF-1α stabilization prevents CD4^+^ T cell activation.

**Figure 4:**
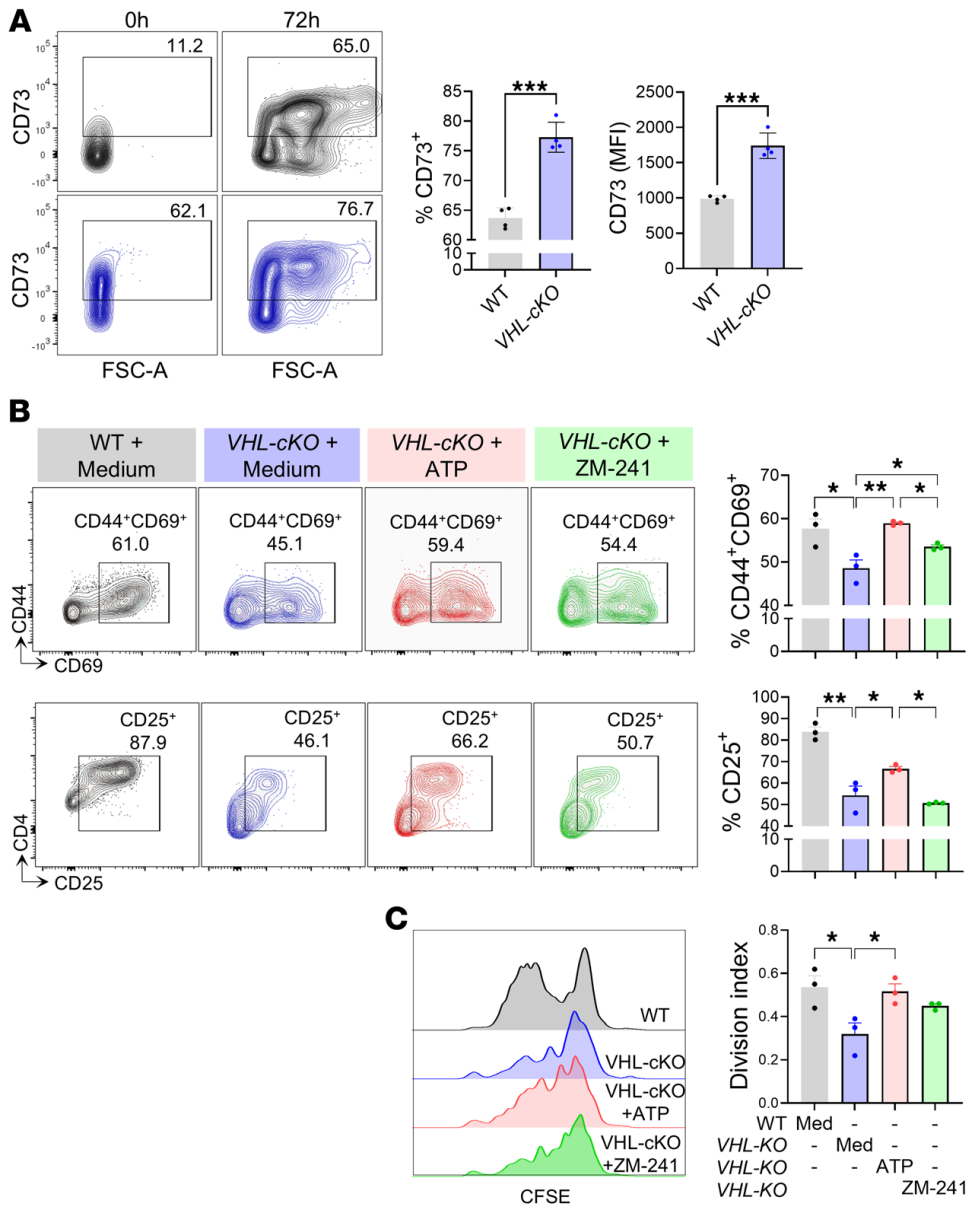
Genetic HIF-1α stabilization prevents CD4^+^ T cell activation. (**A**) Representative contour plots and quantification of CD73 expression levels in *Vhl^+/+^* (WT) (gray) and *Vhl^–/–^* (VHL-cKO) (blue) CD4^+^ T cells 72 hours after TCR activation. (**B** and **C**) Naive VHL-cKO CD4^+^ T cells were cultured with anti-CD3/CD28 antibodies supplemented with ATP (250 nM) or ZM-241385 (1 μM) or maintained in medium alone for 72 hours. (**B**) Contour plots and percentages of cells expressing CD44, CD69, and CD25. (**C**) The proliferation rates were assessed through CFSE dilution 72 hours after activation and visualized as a histogram and a division index. Independent *t* test was performed to compare WT vs. VHL-cKO in **A** (*n* = 4). One-way ANOVA followed by Tukey’s post hoc test was conducted for **B** and **C** (*n* = 3). *P < 0.05, **P < 0.01, ***P < 0.001.

To further explore the underlying mechanisms involved, we used T cells with stabilized HIF-1α obtained from *Vhl*-floxed crossed with *Cd4^cre^* transgenic mice (*VHL-cKO*), and found elevated levels of CD73 compared with those in control cells both before and after TCR stimulation (Figure 4A). Consequently, *VHL-cKO* cells exhibited a pronounced decrease in the expression of activation markers and proliferation. However, the addition of 250 nM ATP successfully reversed such immunosuppressive effects. Furthermore, treatment of VHL-cKO CD4^+^ T cells with the A2aR inhibitor (ZM-241) reversed the frequency of cells expressing the activation markers CD44 and CD69 (Figure 4, B and C). In summary, these data suggest that HIF-1α stimulates CD73 expression, leading to concomitant ADO production, which, in turn, mediates the suppression of the effector response of CD4^+^ T cells.

### T. cruzi infection induces HIF-1α stabilization in T cells from target tissues.

**Figure 5:**
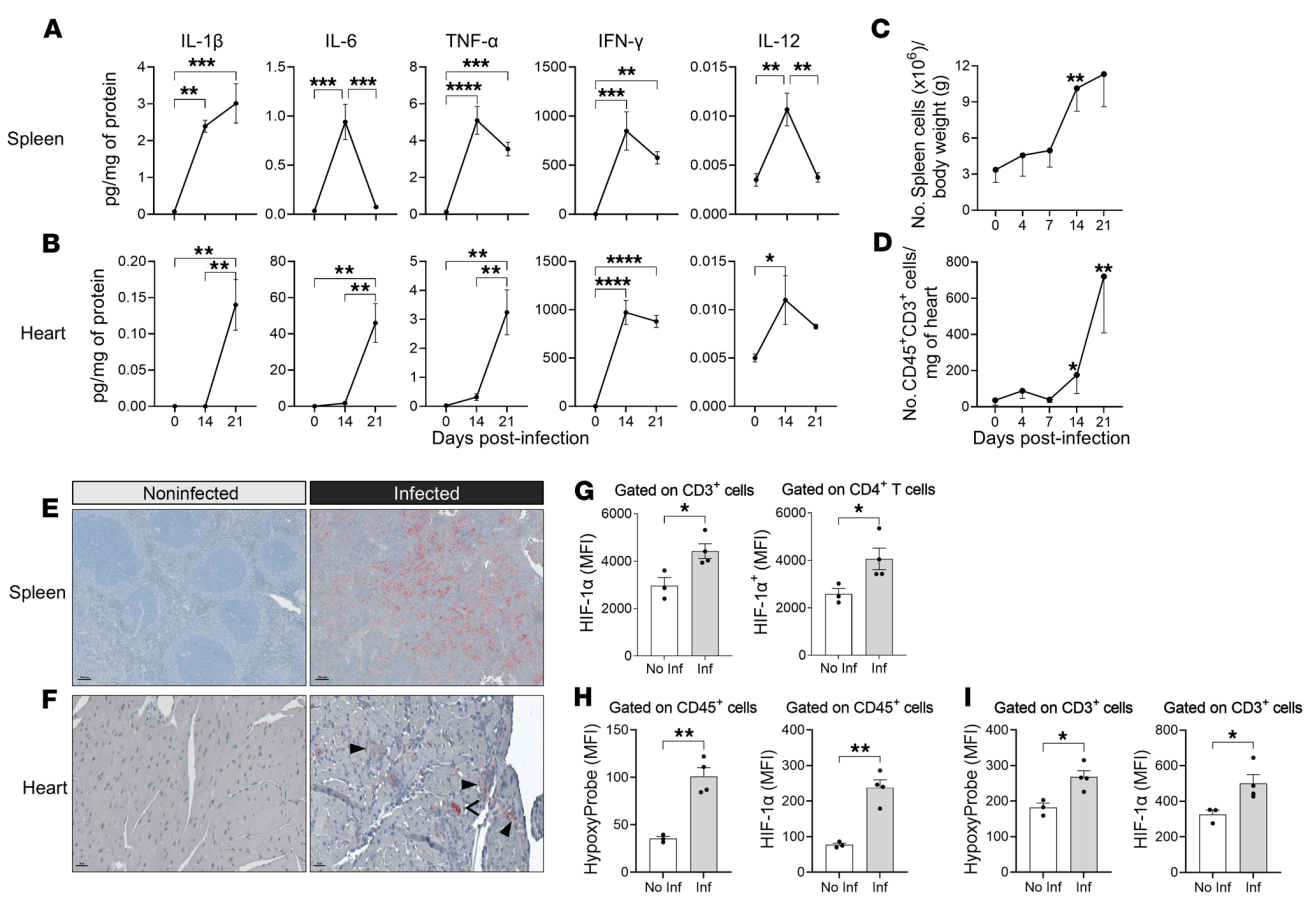
*T.cruzi* infection induces HIF-1α stabilization in T cells from target tissues. C57BL/6J (WT) mice were infected with *T. cruzi*, and target tissues were obtained at the indicated time points after infection. (**A** and **B**) Inflammatory cytokine levels in the spleen (**A**) and cardiac (**B**) tissue of individual mice (*n* = 4 per time) at 0, 14, and 21 days postinfection (dpi) were measured by bead-based immunoassays and normalized to the total protein concentration. (**C** and **D**) The number of total spleen cells normalized to body weight (**C**) and the number of CD45^+^CD3^+^ cells in cardiac tissue normalized to tissue weight (**D**) at 0, 4, 7, 14, and 21 dpi (*n* = 5 per time). (**E** and **F**) Pimonidazole staining of spleen (**E**) or cardiac (**F**) tissue sections from noninfected and infected mice (17 dpi). Representative images of spleen (original magnification, ×7) and heart (original magnification, ×30) are shown (brown, pimonidazole adducts; arrowheads, infiltrating immune cells; open arrowhead, amastigote niche). Scale bars: 100 µm (**E**), 30 µm (**F**). (**G**) Expression of HIF-1α in CD3^+^ and CD4^+^ T cells from noninfected (No Inf, *n* = 3) and infected (14 dpi) (Inf, *n* = 4) spleens measured by flow cytometry. (**H** and **I**) MFIs of pimonidazole adduct (Hypoxyprobe) (**H**) and HIF-1α (**I**) expression in CD45^+^ and CD3^+^ cells in cardiac tissue from noninfected (No Inf, *n* = 3) and infected (17 dpi) (Inf, *n* = 4) mice measured by flow cytometry. One-way ANOVA followed by Tukey’s post hoc test was conducted for **A** and **B**. Independent *t* test was conducted for **C**, **D**, and **G**–**I**. *P < 0.05, **P < 0.01, ***P < 0.001, ****P < 0.0001.

To explore the interplay between the host immune response and HIF-1α expression, we used an in vivo model involving mice infected with *T. cruzi* parasites. Given the significant influence of signals from the microenvironment on activation and effector immune responses (31), we characterized the kinetics of inflammatory cytokines after infection. In comparison with noninfected mice, spleen tissues from infected mice exhibited elevated levels of IL-1β, TNF-α, and IFN-γ at 14 days postinfection (dpi), which persisted until 21 dpi. Additionally, there was a peak in IL-6 and IL-12 at 14 dpi, followed by a decrease at 21 dpi (Figure 5A). Consistent with prior findings (32), cardiac tissue displayed increased IFN-γ and IL-12 levels at 14 dpi. In contrast, the levels of IL-1β, IL-6, and TNF-α peaked at 21 dpi (Figure 5B). These higher cytokine levels coincided with an increased number of immune cells in the spleen (Figure 5C) and T cells in the heart (Figure 5D).

The rise in the number of immune cells and the development of an inflammatory environment can induce hypoxia. Using pimonidazole staining, we assessed the presence of hypoxia in infected target tissues. In contrast to the noninfected spleen, which exhibited a conserved structure without pimonidazole deposits, the infection-induced tissue disorganization was accompanied by substantial foci of hypoxia (Figure 5E). Similarly, noninfected cardiac tissue exhibited neither immune infiltration foci nor tissue hypoxia. However, infection led to a reduction in O_2_ levels throughout the tissue, with a notable emphasis on the foci of immune infiltrates and amastigote niches (Figure 5F).

Considering that different environmental cues trigger HIF-1 signaling in immune cells, we examined HIF-1α expression in T cells following infection. In infected spleens, the expression of this transcription factor was greater in CD4^+^ T cells than in noninfected mice (Figure 5G). Additionally, we found that pimonidazole (Hypoxyprobe) staining and HIF-1α expression increased in leukocytes (CD45^+^) and T cells (CD3^+^) from cardiac tissue after infection (Figure 5, H and I). These findings suggest that *T. cruzi* infection induces the stabilization of HIF-1α in splenic and cardiac T cells.

### T. cruzi infection expands the CD39^+^CD73^–^P2X7^+^ CD4^+^ T cell population.

**Figure 6:**
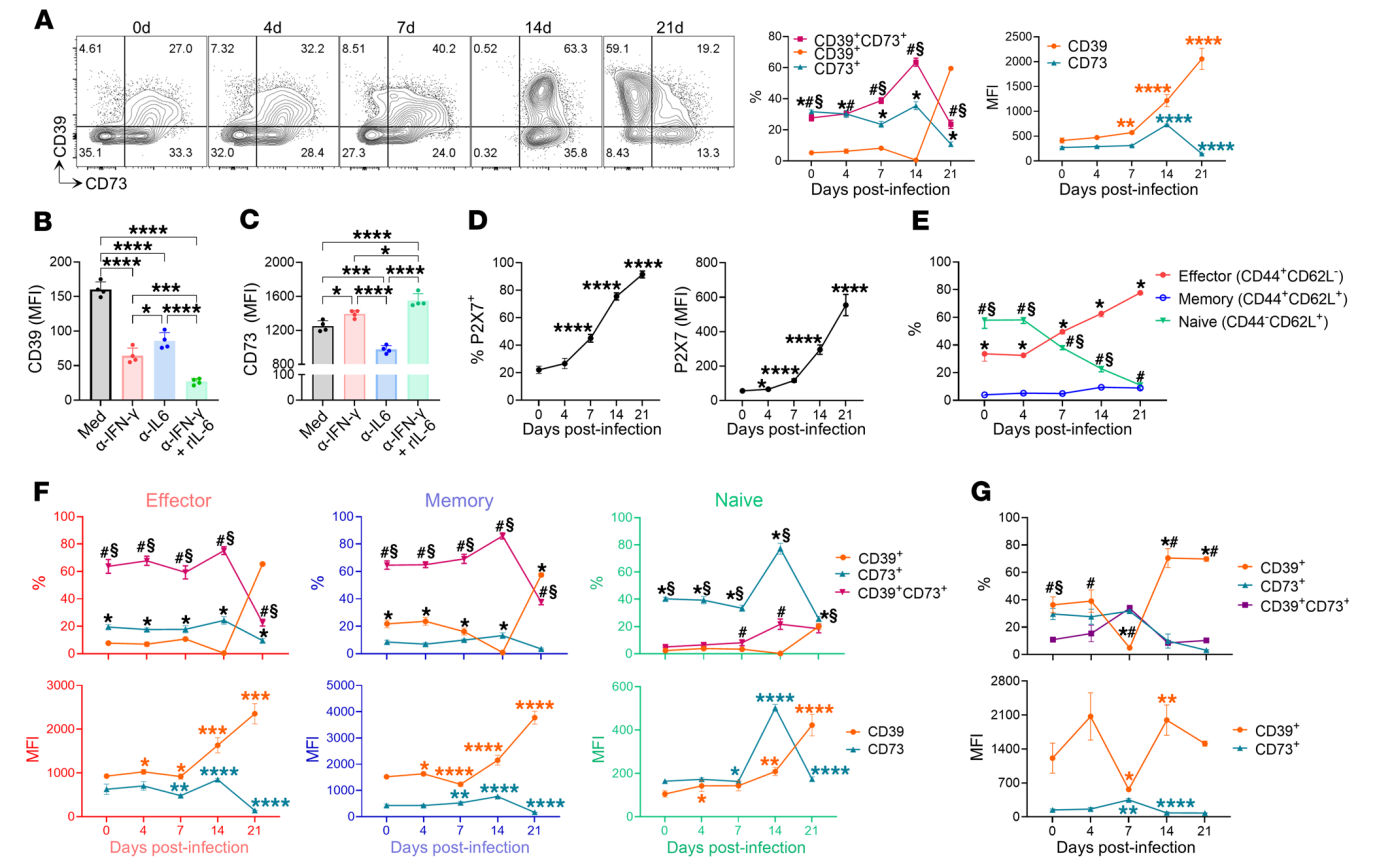
*T.cruzi* infection expands a CD39^+^CD73^–^P2X7^+^ CD4^+^ T cell population. (**A**) Representative contour plots and quantification of CD39 and CD73 expression in splenic CD4^+^ T cells at the indicated time points after infection (*n* = 5 per time). (**B** and **C**) CD4^+^ T cells were TCR-stimulated in the presence of neutralizing anti–IFN-γ (40 μg/mL) or anti–IL-6 (5 μg/mL) antibodies, recombinant IL-6 (10 pg/mL), or isotype control. MFI of CD39 (**B**) and CD73 (**C**) was evaluated at 72 hours after activation by flow cytometry. (**D**) P2X7 expression in splenic CD4^+^ T cells at the indicated time points. (**E**) Kinetics of effector memory (CD44^+^CD62L^–^), central memory (CD44^+^CD62L^+^), and naive (CD44^–^CD62L^+^) CD4^+^ T cell population frequency at the indicated time points after infection. (**F**) Frequency (top) and MFIs (bottom) of CD39 and CD73 expression in effector, memory, and naive CD4^+^ T cell populations. (**G**) Kinetics of CD39 and CD73 expression in cardiac CD45^+^CD3^+^CD4^+^ cells displayed as frequency (top) and MFIs (bottom). Two-way ANOVA followed by Tukey’s post hoc test was performed to compare frequencies in **A**–**G** (*CD39^+^ vs. CD73^+^, ^#^CD39^+^ vs. CD39^+^CD73^+^, ^§^CD73^+^ vs. CD39^+^CD73^+^; *P* < 0.05) (*effector vs. memory, ^#^effector vs. naive, ^§^memory vs. naive; *P* < 0.05). Independent *t* tests were performed to compare each time point with the previous one. One-way ANOVA followed by Tukey’s post hoc test was conducted for **B** and **C**. *P < 0.05, **P < 0.01, ***P < 0.001, ****P < 0.0001.

*T. cruzi* infection leads to chronic disease, which is linked to insufficient immune response and failure to completely clear the parasite. Whether CD39 and CD73 expression by T cells could be implicated in the parasite persistence was evaluated. We next investigated the kinetics of CD39 and CD73 ectoenzyme expression on CD4^+^ T cells at various time points after infection using the gating strategy depicted in Supplemental Figure 3A. In control spleens, a greater frequency of CD73^+^ cells, along with CD39^+^CD73^+^ coexpression, was observed, while the frequency of CD39^+^ cells was relatively low in CD4^+^ T cells. The CD39 levels and the frequency of double-positive CD39^+^CD73^+^ CD4^+^ T cells increased after *T. cruzi* infection, prevailing over single-positive populations, peaking at 14 dpi. However, at 21 dpi, CD73 expression abruptly decreased, and the CD39^+^CD73^–^ phenotype became predominant (Figure 6A). This pattern was also consistent for blood-derived CD4^+^ T cells (data not shown). These findings reinforce the idea that CD73 is the rate-limiting ectoenzyme in purinergic metabolism (33).

The abrupt decline in CD73 expression was accompanied by low levels of IL-6 and high levels of IFN-γ in cardiac (14 dpi) and splenic (21 dpi) infection. To gain further insight into the factors underlying the diminution of CD73 expression, CD4^+^ T cells were activated and incubated with blocking anti–IL-6 antibodies or with anti–IFN-γ alone or combined with recombinant IL-6 (Figure 6, B and C). IFN-γ neutralization increased CD73 expression, an effect boosted by the addition of recombinant IL-6. Conversely, blocking IL-6 led to a significant decrease in CD73 levels. These findings suggest that low levels of IL-6 concomitant with high levels of IFN-γ are involved in decreased CD73 expression during T cell activation.

The *T. cruzi* infection robustly induced P2X7R expression, with nearly all cells expressing P2X7 at 21 dpi, highlighting the importance of eATP signaling in infection control (Figure 6D).

Next, we examined the expression of CD39 and CD73 ectoenzymes within naive, central memory (CM), or effector memory (EM) CD4^+^ T cells. While naive (CD44^lo^CD62L^+^) CD4^+^ T cells predominated in noninfected spleens (58.0% ± 6.0% of naive, 33.6% ± 5.2% of effector, and 4.0% ± 0.4% of memory), the EM (CD44^hi^CD62L^–^) population expanded after infection, becoming predominant at 7 dpi and further increasing until 21 dpi (11.0% ± 1.1% of naive, 77.7% ± 1.1% of effector, and 8.9% ± 1.1% of memory). Although the CM (CD44^hi^CD62L^+^) population also expanded, it remained the least predominant at all evaluated time points (Figure 6E). While in EM and CM populations, CD39 and CD73 coexpression was consistently greater than in the single-positive populations, naive cells exhibited predominantly CD73 expression at all times analyzed. Interestingly, CD39 expression substantially increased, and CD73 decreased across all subsets by 21 dpi (Figure 6F).

In cardiac tissue, before infection, the frequencies of CD39^+^ and CD73^+^ CD4^+^ T cells were similar, with relatively low coexpression levels (36.3% ± 11.6% for CD39^+^, 29.6% ± 8.2% for CD73^+^, and 10.8% ± 1.6% for CD39^+^CD73^+^). However, after infection, there was a notable shift in CD39 expression dynamics. At 7 dpi, CD39 expression dropped, but at 14 dpi, approximately 70% of the CD4^+^ T cells were CD39^+^, which persisted until 21 dpi. This increase coincided with marked T cell accumulation in the cardiac tissue. While CD39 expression exhibited these dynamic changes, CD73 expression remained low at all evaluated time points (Figure 6G). Importantly, FoxP3^+^ Tregs in infected cardiac tissue at 21 dpi constituted 3.9% ± 1.8% of CD45^+^CD4^+^ T cells.

These results support the hypothesis that CD39 upregulation on T cells may regulate microbicidal activity by limiting eATP availability, whereas low levels of CD73 may mitigate the tissue repair and cardioprotective (34) effects of eADO in response to infection.

### ATP potentiates and CD73 activity regulates the anti–T. cruzi cytotoxic CD4^+^ T cell capacity.

**Figure 7:**
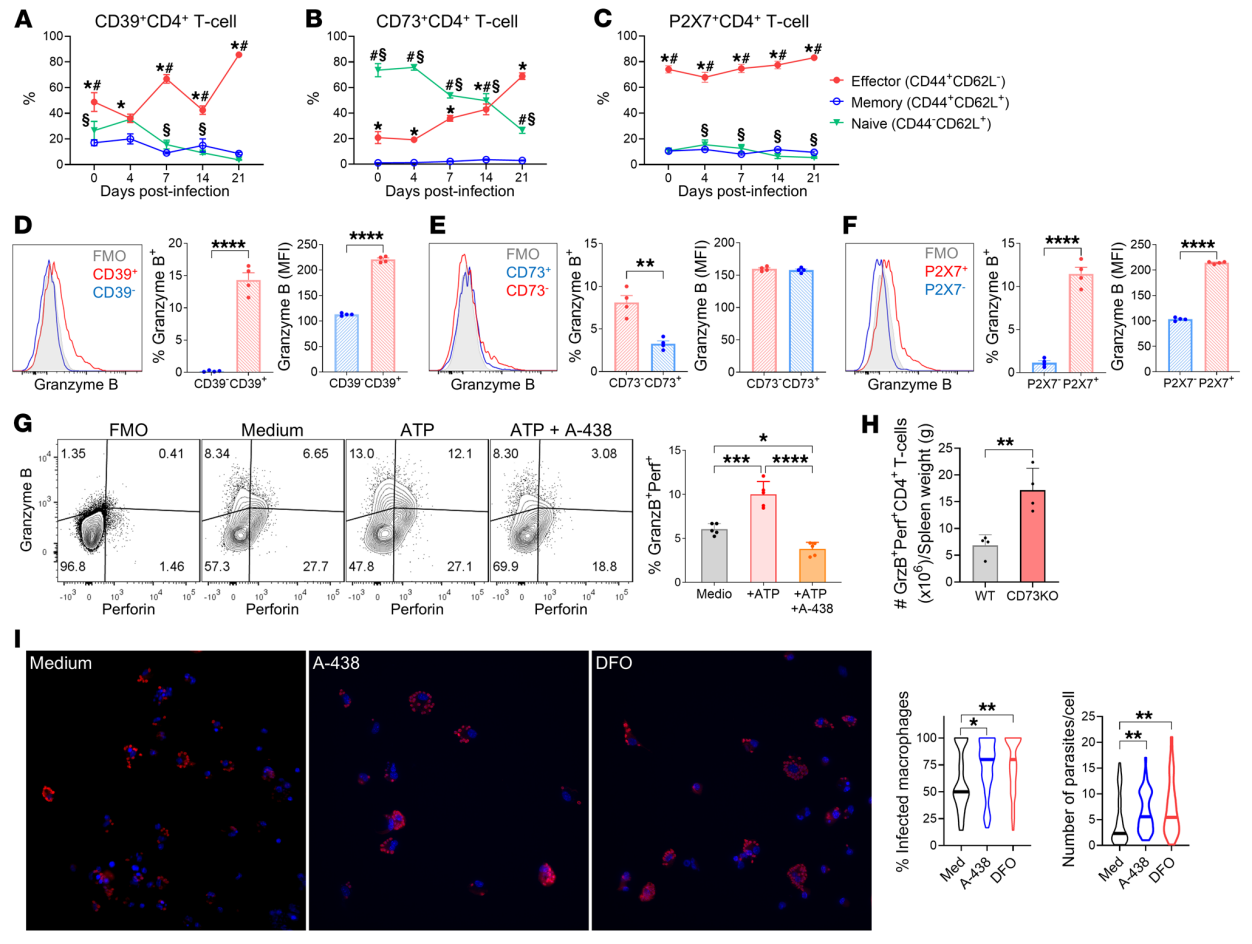
ATP potentiates and CD73 activity regulates the anti–*T.* *cruzi* CD4^+^ T cell–induced microbicidal capacity. (**A**–**C**) Frequencies of effector, memory, and naive cells in CD39^+^ (**A**), CD73^+^ (**B**), and P2X7^+^ (**C**) spleen CD4^+^ T cell compartments at the indicated time points (*n* = 5 per time). (**D**–**F**) Granzyme B expression in CD39^+^ and CD39^–^ (**D**), CD73^+^ and CD73^–^ (**E**), and P2X7^+^ and P2X7^–^ (**F**) splenic CD4^+^ T cells at 14 dpi (*n* = 4 per group). (**G**) Representative contour plot and quantification of granzyme B^+^perforin^+^ (GranzB^+^Perf^+^) expression in isolated CD4^+^ T cells from 14-dpi spleens that were cultured with ATP (100 μM) or ATP plus A-438 (25 μM) or maintained in medium alone for 16 hours (*n* = 5 per group). (**H**) Relative number of GranzB^+^Perf^+^CD4^+^ T cells in the spleens of *Nt5e^–/–^* (CD73-KO) and *Nt5e^+/+^* (WT) mice (*n* = 4 per group). The absolute numbers were normalized to the spleen weight. (**I**) Sorted spleen CD4^+^ T cells from 14-dpi mice were treated with A-438 (25 μM) or DFO (5 μM) and cocultured with in vitro–infected macrophages. The frequency of infected macrophages and the number of parasites per cell were evaluated through high-throughput microscopy using an unsupervised method. Representative images are shown (original magnification, ×40; blue, nuclei; red, *T. cruzi* amastigotes). Two-way ANOVA followed by Tukey’s post hoc test was conducted for **A**–**C** (*effector vs. memory, ^#^effector vs. naive, ^§^memory vs. naive; *P* < 0.05). Independent *t* tests were conducted for **D**–**F** and **H**. One-way ANOVA followed by Tukey’s post hoc test was conducted for **G** and **I**. *P < 0.05, **P < 0.01, ***P < 0.001, ****P < 0.0001.

Next, we sought to examine the expression of CD39, CD73, and P2X7R in the CD4^+^ T cell populations during *T. cruzi* infection. EM CD39^+^ CD4^+^ T cells were always dominant, peaking at 21 dpi (Figure 7A). In contrast, the CD73^+^ population was largely composed of naive CD4^+^ T cells until 14 dpi, after which EM cells became predominant (Figure 7B). Notably, approximately 80% of the P2X7R^+^ population were EM cells (Figure 7C).

While cell-mediated cytotoxicity has traditionally been attributed to CD8^+^ T cells, recent studies have highlighted the role of cytotoxic CD4^+^ T cells in combating intracellular pathogens and tumors (21, 35). Given the observed dynamics, we propose that eATP metabolism might influence the effector response mediated by CD4 CTLs in *T. cruzi* infection control. We found that the frequency and expression levels of the cytotoxic molecule granzyme B were increased in CD39^+^CD73^–^P2X7R^+^ CD4^+^ T cells (Figure 7, D–F).

Regarding CD4 CTL development, some authors consider them as functional variants of Th1 cells; there is also evidence that CD4 CTLs might constitute other CD4^+^ T cell subsets or even a heterogeneous population congregating cytolytic lymphocytes that originate from different T cell subsets, such as Tregs, Th1 cells, and Th2 cells (22). To clarify this point, we analyzed the transcription factors and the class I–restricted T cell–associated molecule (CRTAM) previously linked to the CD4 CTL profile (22, 36) in the context of *T. cruzi* infection. In our experimental model, at the peak of infection (14 dpi), the vast majority of splenic granzyme B^+^ and perforin^+^ CD4^+^ T cells expressed CRTAM. Furthermore, about 51% of granzyme B^+^ and 45% of perforin^+^ CD4^+^ T cells expressed T-bet, and about 22% of granzyme B^+^ and 17% of perforin^+^ CD4^+^ T cells expressed Eomes (Supplemental Figure 3, B–D). Although T-bet and Eomes are transcription factors associated with CD4 CTL biology, in agreement with our results, only 12.9% of CRTAM^+^ CD4^+^ T cells express Eomes in sorted murine spleen cells after anti-CD3 and anti-CD28 stimulation and 5 days in the presence of IL-2. Ectopic expression of CRTAM in vivo induces CD4 CTL differentiation (36). Further studies are needed to fully elucidate the relationship between Eomes, T-bet, and the expression of cytotoxic molecules, as well as their interplay with CRTAM in CD4^+^ T cells, in the context of *T. cruzi* infection.

FoxP3^+^ cells accounted for less than 5% of the total CD4^+^ T cell population and exhibited negligible levels of granzyme B and perforin expression (Supplemental Figure 3E), indicating that Tregs are unlikely to mediate the expression of granzyme B and perforin during the infection.

To further elucidate the role of eATP signaling, spleen CD4^+^ T cells from 14 dpi mice were incubated with ATP (100 μM) or the P2X7R inhibitor A-438. The addition of ATP expanded the population expressing granzyme B and perforin, compared with cells cultured in medium alone. In agreement, treatment with A-438 revealed that the ATP-mediated increase of double-positive population was through the engagement of the P2X7R. Furthermore, P2X7R inhibition significantly reduced the population with CTL phenotype compared with untreated cells, suggesting that ATP released by activated cells has stimulatory, paracrine-type effects (Figure 7G). Moreover, the number of granzyme B^+^perforin^+^ CD4^+^ T cells was higher in spleen from *Cd73^–/–^* than in spleen from WT mice (Figure 7H), highlighting the involvement of purinergic metabolism in the differentiation of CD4^+^ T cells with cytotoxic potential.

To explore more deeply the role of P2X7R signaling in T cell effector function, isolated splenic CD4^+^ T cells from infected mice were treated with the P2X7R inhibitor A-438 and cocultured with in vitro–infected macrophages. Inhibition of the P2X7R on T cells altered the anti–*T. cruzi* response, increasing the percentage of infected macrophages and the number of parasites per cell in comparison with untreated T cells. Similarly, HIF-1 stabilization in CD4^+^ T cells through DFO treatment impaired parasite control (Figure 7I). To assess whether the killing capacity of CD4^+^ T cells depends on cytotoxic effector molecules, we performed a cytotoxicity assay in the presence of concanamycin A, a specific perforin inhibitor. Supplemental Figure 3F shows the specific lysis of primed macrophages — loaded with total trypomastigote protein extract — cocultured with CD4^+^ T cells isolated from the spleen of infected mice. Pretreatment of lymphocytes with concanamycin A significantly reduced the rate of lysis, indicating that CD4^+^ T cells exert perforin-dependent cytotoxicity. Moreover, this treatment with APCP indicated that CD73 may regulate perforin-independent mechanisms, such as Fas-FasL interactions (21, 37) or cytokine-dependent pathways.

### Cardiac explants from patients with CCC have enriched HIF-1 and purine metabolism pathways.

Substantial progress has been made in understanding CCC, but the mechanisms underlying parasite persistence in human cardiac tissue remain elusive. The pathogenesis of heart disease derives from the inability of the immune system to efficiently control infection and cardiac lesions that depend on a prolonged immune response (38). To further explore whether purinergic signaling contributes to the pathogenesis of CCC in humans, we aimed to evaluate gene expression in myocardial tissue obtained from an RNA-Seq dataset conducted by Brochet and collaborators (39), which included samples from 8 end-stage CCC patients (5 females and 3 males) and 6 male control donors.

**Figure 8:**
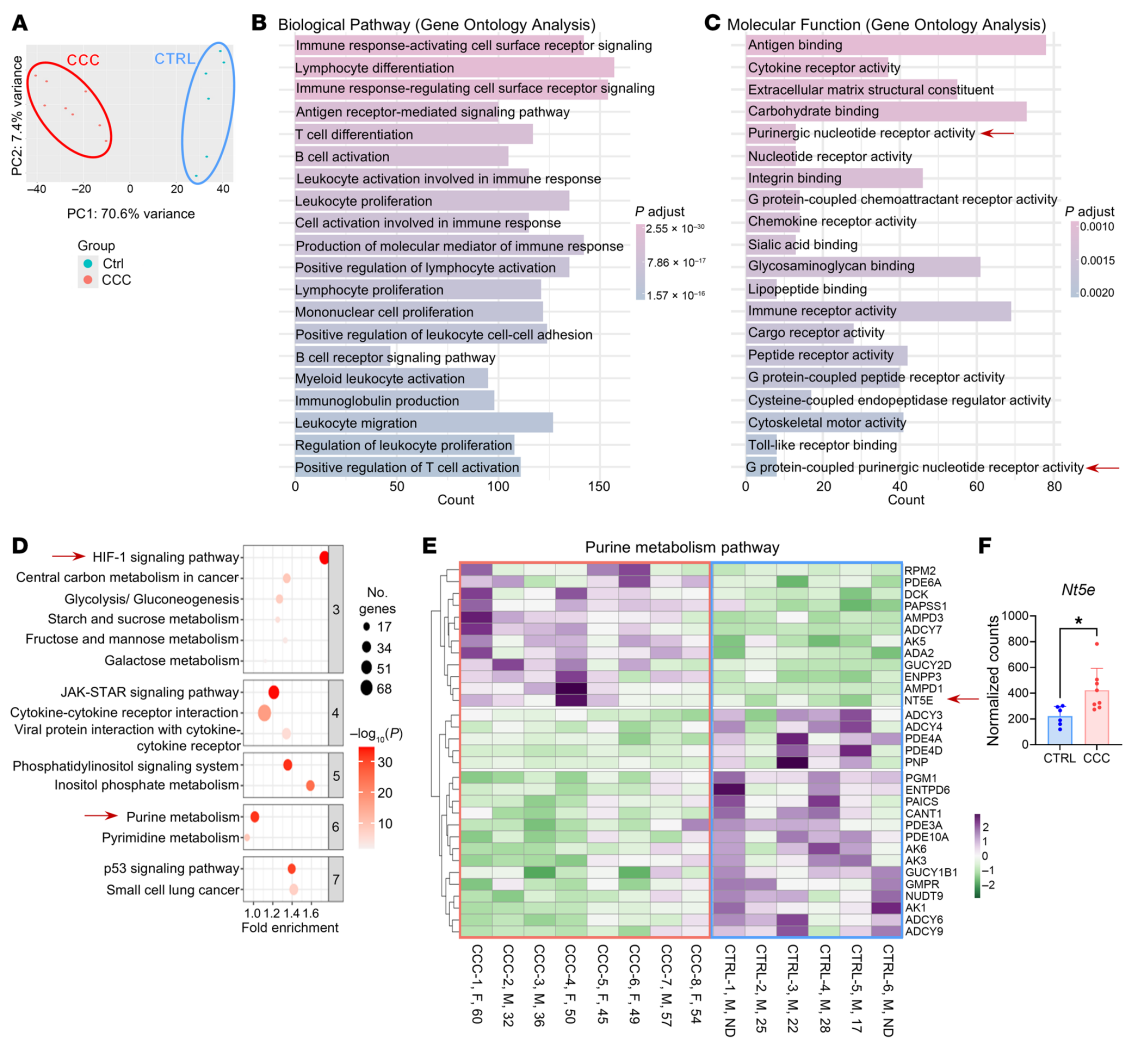
Cardiac explants from patients with CCC have enriched HIF-1 and purine metabolism pathways. Public RNA-Seq data (GSE191081) were obtained from myocardial tissue of patients with end-stage CCC (*n* = 8) and healthy donors (CTRL, *n* = 6) (**A**) Principal component analysis of differentially expressed genes (DEGs) between CCC and CTRL. (**B** and **C**) Gene Ontology biological pathway and molecular function enrichment analysis of upregulated genes in CCC. (**D**) Hierarchical pathway enrichment analysis of DEGs in CCC compared with control samples. (**E**) Heatmap of genes involved in the purine metabolism pathway in CCC and CTRL. The sex and age of each patient is indicated (F, female; M, male). (**F**) Normalized counts of the *Nt5e* gene were compared using an independent-samples *t* test. *P < 0.05.

Principal component analysis of differentially expressed genes highlighted substantial differences between CCC samples and controls (Figure 8A). Gene Ontology analysis revealed an enrichment of biological pathways associated with immune activation in CCC samples, particularly those involving T cell immune responses (Figure 8B). Furthermore, these patients exhibited enriched molecular function pathways associated with the activity of purinergic nucleotide receptors and G protein–coupled purinergic receptors, including ADO receptors (Figure 8C). To delve deeper into these findings, we performed a pathway enrichment analysis, which notably identified the HIF-1 signaling pathway and purine metabolism among of the top 8 most enriched pathways (data not shown). Additionally, hierarchical clustering revealed that the HIF-1 signaling pathway was grouped among the first 3 most overrepresented clusters, while purine metabolism was clustered in the sixth cluster (Figure 8D). Heatmap analysis of the enriched purine metabolism pathway revealed distinct differences in expression patterns between CCC patients and controls (Figure 8E). Notably, among this set of transcripts, the expression of *Nt5e*, encoding the CD73 protein, was upregulated in CCC samples (), but CD39 gene expression did not show differences. Importantly, neither the sex nor the age of the patients influenced these analyses. These findings underscore the participation of the purinergic system and HIF-1 signaling in CCC progression in humans.

## Discussion

In the present study, we comprehensively investigated the role of the HIF-1 signaling pathway and CD39/CD73/ADO axis in regulating the effector responses of CD4^+^ T cells upon activation, as in the context of *T. cruzi* infection. We have shown associations of ectonucleotidases with parasite persistence. The purinergic signaling system plays dualistic roles. It promotes the activation of the immune responses necessary for pathogen elimination while also triggering suppressive mechanisms to mitigate collateral damage, which can result in excessive healing with scarring (40, 41). Hypoxia, as a modulator of this system, has varying influence over T cell effector functions (42). Hence, the participation of HIF-1, in concert with purinergic signaling, in T cell activity needs to be further investigated. We demonstrate that there is a substantial accumulation of HIF-1α protein following TCR stimulation, reinforcing the notion that immune cells can stabilize HIF-1 under normoxic conditions. HIF-1α expression is concomitant with the upregulation of CD39, CD73 ectoenzymes, and P2X7, A2a, and A2b receptors. The results strongly suggest that activation of the purinergic system is a key step following TCR engagement.

Our data indicate that eATP affects CD4^+^ T cell activation in a concentration-dependent manner, in concordance with Trabanelli and colleagues (28). Strikingly, we found that ATP at concentrations found in tumor (43) and infectious (32) environments inhibits anti-CD3/CD28–stimulated CD4^+^ T cells from noninfected control mice. Conversely, cells obtained from infected mice respond to the highest concentration of ATP, displaying an increased frequency of CD4^+^ T cells with CTL effector molecules. We also determined that P2X7R is responsible, at least in part, for the ATP-mediated response. Overall, these findings demonstrate the interplay between eATP concentrations and cell activation status and establish that P2X7 is a key receptor that supports activation of CD4^+^ T cells with cytotoxic potential.

Considering that TCR engagement upregulates ectoenzyme expression, leading to the breakdown of ATP to ADO, we investigated whether CD73 activity regulates the T cell response. Abrogation of CD73 activity was associated with enhanced T cell activation (44) and the expression of cytotoxic effector molecules. We further observed that ADO-mediated signaling through the A2aR attenuates CD4^+^ T cell microbicidal response. Although it is generally accepted that A2aR activation promotes the differentiation of T cells into Tregs (45), a very low percentage of FoxP3^+^ cells or cells producing IL-10 were present in the cultures, indicating that the observed effect was mainly affecting conventional CD4^+^ T cells. Results provide evidence that the balance of P2X7 and A2a receptors controls the magnitude of purinergic signaling sensed by CD4^+^ T cells with cytotoxic potential and suggest that ADO is a central mediator in regulating their effector functions.

HIFs play a critical role in maintaining the proper function of a range of immune cell populations. However, the impact of HIF-1 on the differentiation and function of different T cell subsets remains controversial (42). Constitutive HIF-1 activity has been shown to potentiate effector functions in CD4^+^ and CD8^+^ T cells in tumor and viral infections (3, 46). In contrast, other studies have shown that hypoxic conditions impair T cell effector functions (47, 48). In a recent report, Liu and colleagues demonstrated that HIF-1α stabilization worsens CD4^+^ T cell response in *Mycobacterium tuberculosis* murine infection. Interestingly, CD73 was among the 14 most upregulated of 3,524 genes in *Vhl*-cKO compared with WT CD4^+^ T cells from infected lungs (49). In agreement, we found that HIF-1 was associated with increased CD73 activity and decreased amounts of CD4 CTL effector molecules. As expected, this effect was partially reversed by CD73 inhibition. Overall, these results suggest that HIF-1 signaling subverts CD4^+^ T cell effector functions by mediating the production of eADO through CD73 activity. Recent evidence suggests that HIF-2 restrains Th1 response under inflammatory conditions (50), demonstrating complementary roles with HIF-1 in T cells. However, further investigation into the specific mechanisms by which HIF-2 regulates CD4^+^ T cells is essential to fully elucidate its impact.

Our group has examined the relationships between *T. cruzi* infection and purinergic signaling in Chagas disease patients and in murine infection models in depth (24, 25, 32, 51, 52). Although the complete immunological mechanisms involved in the progression of CCC remain elusive, it is widely accepted that the development of cardiac lesions relies on sustained inflammatory responses as a consequence of *T. cruzi* persistence (38). Our results show a robust increase in the number of T cells and Th1 cytokines, associated with prominent areas of hypoxia, and T cells expressing HIF-1α in *T. cruzi*–infected spleen and myocardial tissue. The parasite can modulate the levels of eATP and ADO by directly acting on these molecules (53) or by inducing the expression of ectonucleotidases in immune cells. Strikingly, *T. cruzi* infection promotes the expansion of the CD4^+^ T cell population characterized by a CD39^+^CD73^+^ phenotype. After the peak of parasite load (32, 54), CD39 frankly increased, and CD73 expression was abolished. Another relevant finding of the present work was to establish the pattern of ectoenzyme expression in each CD4^+^ T cell–specialized population: naive, EM, and CM. Our findings suggest that CD39 upregulation may attenuate local microbicidal activity by diminishing eATP availability and allowing parasite persistence. In turn, CD73 downregulation may inhibit the antiinflammatory effect mediated by eADO signaling. The final balance led to a permissive environment for parasites with impaired wound healing that fostered the development of chronicity.

Considering that the observed switch was concomitant with high levels of IFN-γ and low levels of IL-6 in the spleen and heart tissue, we postulate that these two cytokines could be involved in driving the dynamic of expression. Although further work is required to find out the factors involved in determining this kinetics (55), we propose that reduced IL-6 levels and persistently elevated IFN-γ levels may be involved in the rise of CD39 and decrease of CD73 expression. However, it is important to stress that other mechanisms could be involved in decreasing CD73, such as the release by phospholipases or in vesicles (56), as was reported as an intrinsic mechanism to decrease inflammation.

Our study reveals that TCR activation induces the CD4 CTL phenotype. Indeed, we found that CD4^+^ T cells from *T. cruzi*–infected mice exhibit potential perforin-dependent cytotoxic capacity, in agreement with a previous report (21). Comparing our results using the Tulahuen strain, infection with Y strain induces a higher frequency of CD4^+^ T cells with CTL phenotype (21). This apparent discrepancy could be due to differences in replication rates, tissue tropism, and inflammatory profiles (57), which can influence the differentiation and expansion of CD4^+^ T cells with cytotoxic capacity.

Notably, CD39^+^ T cells produced higher effector molecule levels than their CD39^–^ counterparts, and the absence of CD73 on CD4^+^ T cells was associated with increased granzyme B production. Although these observations require further investigation, it is evident that the phenotype of ectoenzyme expression is linked to the production of cytotoxic effector molecules in CD4^+^ T cells.

Few studies have characterized the hypoxic signature of *T. cruzi* infection (58). Building on the premise that HIF-1–mediated signaling affects CD4^+^ T cell activation and may be linked to ATP release, we observed defective parasite control upon HIF-1 stabilization in isolated CD4^+^ T cells. Moreover, ATP stimulation resulted in double-positive granzyme B^+^perforin^+^ CD4^+^ T cell expansion through P2X7R signaling and improved anti–*T. cruzi* response. To our knowledge, our study provides the first evidence linking ATP/P2X7R signaling to the potential cytotoxic capacity of CD4^+^ T cells in controlling parasite infection. These findings highlight the immunoregulatory role of HIF-1, suggesting that manipulating HIF-1 or purinergic signaling could offer significant therapeutic benefits in promoting pathogen clearance and preventing chronic disease. ADO pathway inhibitors may reinvigorate the immune cell effector functions and improve immune response fitness (59). Some strategies of therapeutic interventions that target the HIF and ADO signaling pathways are already in advanced clinical trials (60). In the context of a systemic and intracellular infection, however, our findings evidence the complex dynamics of ectoenzymes and purinergic microenvironment, which represents a significant challenge for therapy.

Data reuse from existing datasets is playing a critical role in advancing biomedical research by allowing the testing of new hypotheses without duplicating data collection efforts. Our analysis of an existing transcriptomic dataset (39) revealed that HIF-1α and purinergic metabolism pathways are among the most differentially expressed pathways and that the CD73-encoding gene *Nt5e* is upregulated in CCC samples, suggesting that the HIF-1– and CD73-induced ADO signaling pathway is overrepresented in *T. cruzi*–infected hearts. In agreement, Cunha-Neto and colleagues reported the upregulation of HIF-1 signaling signature in cardiac tissue from CCC patients compared with non–Chagas disease patients and control donors (61). Notably, the CD39-encoding gene is not overexpressed in comparison with control.

Hypoxia and HIFs regulate T cell immunity, although the effect might differ in acute versus chronic inflammation. Indeed, during the acute stage of *T. cruzi* infection, HIF-1 and CD39 are upregulated, and CD73 is downregulated to foster parasite replication. However, in chronicity, both HIF-1 and CD73 are upregulated, supporting ADO-mediated cardioprotection (62, 63). Consistent with these findings, increased plasma ADO levels are detected in patients with general heart failure (64). Similarly, ADO plays a tissue-protective role in acute inflammatory injuries (65). However, chronically elevated ADO can become detrimental by promoting tissue injury and fibrosis (66). Under these conditions, CD73/ADO signaling is emerging as a critical pathway and therapeutic target in various disorders (67).

The results presented in our study show that the inflammatory environment generated by the immune response to *T. cruzi* in a model of Chagas disease drives the immune cell activity of CD39 and CD73. This is of translational importance, as intense studies are currently being conducted to postulate purinergic signaling as a possible therapeutic target (68). Our results highlight the differential expression of immune cell ectonucleotidases during the infection course, which could hinder the effectiveness of the therapy.

## Methods

### Sex as a biological variable

Gene expression analyses were performed using a previously published dataset (GEO GSE191081), which includes samples from 8 end-stage CCC patients (5 females and 3 males) and 6 male control donors. No sex-related differences were observed among the differentially expressed genes within the CCC group. Therefore, the patient group was analyzed as a whole and compared with the control donors. Sex was not considered as a biological variable in the mouse experiments. Infection experiments were conducted in female mice. Female mice were used to experimentally model infection to ensure consistency with previous studies (24, 69, 70). Sex-based differences in immune responses to *T. cruzi* infection have been reported, with males typically showing higher parasitemia and more severe pathology (71, 72). Experiments not involving infection and involving in vitro studies were conducted in male mice to utilize available counterparts, in line with ethical principles for animal use.

### Mice

C57BL/6J (WT) mice were obtained from Facultad de Ciencias Veterinarias, Universidad Nacional de La Plata, La Plata, Argentina. CD73-deficient mice (CD73-KO; B6.129S1-Nt5e^tm1Lft^/J, JAX stock 018986) were purchased from The Jackson Laboratory.

For T cell–specific deletion studies, mice harboring loxP-flanked *Hif1a* and *Vhl* alleles (49) were bred with transgenic mice harboring the Cre recombinase gene driven by the CD4 promoter (*Cd4^cre^*), resulting in Cre expression during thymocyte development. *Hif1a^fl/fl^* or *Vhl^fl/fl^* littermates were used as controls.

All mice were maintained in a specific pathogen–free unit on a 12-hour light/12-hour dark cycle. The room temperature was maintained at 25°C. The humidity level was controlled between 40% and 60%. The animals had ad libitum access to water and feed in all experiments.

### Human samples

Gene expression profile analysis was performed using the dataset published by Brochet and collaborators (39). The transcripts were from human left ventricular free wall heart tissues of individuals with end-stage heart failure CCC at the time of heart transplantation (CCC patients, 5 females and 3 males). Biopsies from healthy controls (CTRL, 6 males) were from organ donors having no suitable recipient. Details on patient information can be found in Brochet et al. (39), accession number GSE191081 in the Gene Expression Omnibus (GEO) database.

### Experimental infection models

Bloodstream trypomastigotes of *T. cruzi* (Tulahuen strain) were harvested from anesthetized infected mice via heart puncture and maintained for successive passages. For in vivo infection models, 6- to 8-week-old female mice were infected intraperitoneally (i.p.) with 1,000 blood-derived trypomastigotes. Noninfected mice were used as controls. For coculture experiments, blood-derived trypomastigotes were used to infect monolayers of Vero cells. After 7 days, the supernatants were collected and washed twice with PBS.

### Isolation of splenocytes

Spleens were obtained in PBS and passed through a 70 μm nylon cell strainer. Erythrocytes were removed using a lysis buffer (Gibco). Viable cell counts were determined by trypan blue exclusion using a Neubauer chamber.

### Isolation of cardiac immune cells

The isolation of heart leukocytes was carried out as described previously (24). Briefly, hearts were perfused with PBS, weighed, and disaggregated mechanically and enzymatically with 0.25% trypsin solution (MilliporeSigma). The digested tissue was gently pressed through a 40 μm cell strainer. Mononuclear cells and granulocytes were isolated by 35% and 70% bilayer Percoll density gradient centrifugation (GE Healthcare). Viable cell counts were determined by trypan blue exclusion using a Neubauer chamber.

### CD4+ T cell isolation and in vitro activation

Naive (CD44^−^) or total CD4^+^ T cells were obtained by negative selection from splenic single-cell suspensions using magnetic beads (MACS, Miltenyi Biotec, or MojoSort, BioLegend) from 6- to 8-week-old male mice. Then the purified cells were resuspended in RPMI 1640 medium supplemented with 10% FBS, 0.1% gentamicin, and 50 μM β-mercaptoethanol. In 96-well plates, 5 × 10^4^ cells were stimulated with plate-bound anti-CD3 (clone 145-2C11, Invitrogen or BioLegend) and soluble anti-CD28 (clone 37.51, BD Biosciences or BioLegend) antibodies. After 72 hours, the cells were replated with fresh medium supplemented with recombinant IL-2 (20 ng/mL; PeproTech). Under the indicated conditions, ATP, ADO, receptor inhibitors, and other compounds (listed in Supplemental Table 2) were added at the beginning of activation. The cells were cultured at 37°C and 5% CO_2_.

### Cell proliferation assays

Cell proliferation was measured using the cell division tracking dye carboxyfluorescein diacetate succinimidyl ester (CFSE; eBioscience). Purified CD4^+^ T cells were labeled with 5 μM CFSE in PBS for 7 minutes, and the reaction was stopped with 2 mL of ice-cold 10% FBS in PBS to absorb any unbound dye. After washing, the cells were resuspended in a warm complete RPMI medium before being plated in anti-CD3/CD28 antibody–coated plates. After 72 hours of incubation, the cells were stained and subjected to FACS analysis. Unstimulated CFSE-labeled cells served as a non-dividing control.

### Flow cytometry

#### Surface staining.

The cell suspensions were incubated with live/dead stains. Then fluorophore-conjugated antibody cocktails (Supplemental Table 3) were added and incubated for 30 minutes at 4°C. Then the cells were washed with staining buffer (PBS with 5% FBS), resuspended, and stained on an LSRII or Fortessa flow cytometer (BD Biosciences). The data were analyzed using FlowJo software 10 (Tree Star Inc.). The gating strategies used are shown in Supplemental Figure 1A and Supplemental Figure 3B.

#### Intracellular staining.

To evaluate the expression of intracellular molecules, surface-labeled cells were fixed and permeabilized using BD Cytofix/Cytoperm and Perm/Wash (BD Biosciences) or FoxP3 staining buffer (eBioscience) according to the manufacturer’s instructions. Afterward, the cells were incubated with the following antibodies: monoclonal anti–mouse IFN-γ, granzyme B, perforin, Ki-67, Eomes, T-bet, FoxP3, and/or rabbit monoclonal anti–HIF-1α. Finally, the cells were washed and incubated with anti-rabbit Alexa Fluor 488 or Alexa Fluor 647 for 15 minutes. Data were acquired as described above.

### Cell sorting and ex vivo ATP stimulation

Spleen cells were obtained from infected mice (14 dpi) as described above. CD4^+^ T cells were purified from CD11b^–^CD4^+^ cells by cell sorting (FACSAria II, BD Biosciences). A total of 4 × 10^5^ cells were treated with A-438 (25 μM) for 1 hour or allowed to rest in the medium. Then a fresh medium supplemented with ATP (100 μM) was added under the indicated conditions. After 16 hours, the cells were incubated with PMA (50 ng/mL), ionomycin (1 μg/mL), brefeldin, and monensin for 4 hours. Subsequently, the cells were washed and prepared for staining as described above.

### Macrophage and T cell cocultures

Peritoneal cells were collected from uninfected mice by a quick peritoneal lavage method and seeded in adherent plates. After 3 hours of incubation, the nonadherent cells were removed to maintain the attachment of the peritoneal macrophages. The macrophages were infected with *T. cruzi* trypomastigotes for 3 hours. Then the cells were washed with PBS twice and incubated for 48 hours. Sorted CD4^+^ T cells from infected mice (14 dpi) were treated for 1 hour with A-438 (25 μM) or DFO (5 μM) and cocultured with in vitro–infected macrophages (at a ratio of 1:1) for 18 hours.

After incubation, the macrophages were fixed and permeabilized for staining with an anti–*T. cruzi* antibody and immunofluorescent detection. The frequency of infected macrophages and the number of parasites per cell were determined by an unsupervised approach via high-throughput microscopy (IN Cell Analyzer 2500HS, Cytiva).

For the cytotoxicity experiment, peritoneal macrophages were loaded with 30 mg/mL trypomastigote antigen extract (2.5 μM; CFSE^hi^) for 45 minutes or left unloaded (0.5 μM; CFSE^lo^). The cells were washed and cocultured for 18 hours with purified CD4^+^ T cells obtained from infected mice (14 dpi) at a target/effector ratio of 1:8. CD4^+^ T cells were pretreated with the perforin inhibitor concanamycin A (100 nM) either alone or in combination with APCP (100 μM) for 1 hour or left in medium alone. After the exclusion of dead (7-aminoactinomycin D–positive) macrophages, the specific lysis was calculated using the formula: % specific lysis = 100 × [(CFSE^hi^/CFSE^lo^) macrophages alone – (CFSE^hi^/CFSE^lo^) macrophages and T cells] / [(CFSE^hi^/CFSE^lo^) macrophages alone].

### Real-time PCR

RNA was extracted from 1.5 × 10^5^ CD4^+^ T cells before or after in vitro anti-CD3/CD28 stimulation by TRIzol reagent (Invitrogen) and reverse-transcribed into cDNA using a First Strand cDNA Synthesis Kit (Thermo Fisher Scientific) and GeneAmp PCR System 9700 (Applied Biosystems). Transcripts were quantified by real-time quantitative PCR on a 7500 Real-Time PCR System (Applied Biosystems) sequence detector. *Hprt* was used as a control gene to calculate the ΔCt values for independent triplicate samples. The relative amounts of target/Hprt transcripts were calculated using the 2^−ΔΔCt^ method. These values were then used to calculate the fold increase in the expression of specific mRNAs compared with that in non-stimulated cells. The sequences of primers used are displayed in Supplemental Table 1.

### ADO and ATP measurements

In 96-well plates, 5 × 10^4^ CD4^+^ T cells were stimulated with anti-CD3 and anti-CD28 antibodies in 200 μL of RPMI 1640 supplemented medium. ATP and ADO levels were measured in culture supernatants with an ATP Bioluminescence Kit (CS0012) or an ADO Fluorometric Kit (MAK433), respectively, according to the manufacturer’s instructions (Sigma-Aldrich). Briefly, the samples were incubated with a luminescent or fluorometric reaction mix at room temperature for 15 minutes in a 96-well black plate protected from light. Luminescence or fluorescence was measured at 560–580 nm in a Synergy 2 Multi-Mode Reader (BioTek). A standard curve was plotted to calculate the purine concentration, and a regression analysis was applied as indicated by the kit manufacturer.

### Cytokine analyses

Spleen and cardiac lysates were analyzed for IL-1α, IL-1β, IL-6, IL-10, IL-12p70, IL-17A, IL-23, IL-27, CCL2 (MCP-1), IFN-β, IFN-γ, TNF-α, and GM-CSF levels using a bead-based multiplex assay (740446, LEGENDplex) and flow cytometry (FACSCanto II, BD Biosciences), according to the manufacturer’s instructions. The data were analyzed by LEGENDplex software after generation of the standard curves. IL-2 levels were determined using an ELISA Max IL-2 Kit (BioLegend). The cytokine levels were normalized to the total protein concentration determined by the Bradford method (Bio-Rad).

### Cell and tissue hypoxia

Hypoxyprobe-1, a pimonidazole hydrochloride, is reduced by nitroreductases in hypoxic cells (oxygen pressure [pO_2_] < 10 mmHg) to form covalent protein adducts that can then be detected immunohistochemically using a Hypoxyprobe-1 kit (Hypoxyprobe Inc.). Uninfected and infected mice received a single i.p. injection of pimonidazole (60 mg/kg in PBS), and spleens and hearts were harvested after 90 minutes. Cell hypoxia was investigated in single-cell suspensions using a fluorescent anti-pimonidazole antibody and flow cytometry. Tissue hypoxia was visualized by microscopy (PhenoImager Fusion, Akoya Biosciences) as a brown stain following immunohistochemistry by DAB chromogen detection.

### RNA-Seq analyses

#### Differential expression analysis.

Statistical analyses were performed using R (4.3.1). The DESeq2 package (v1.40.2) was used for data normalization and differential gene expression analyses, and the shrinkage function was used to correct the log_2_ fold change (log_2_FC). The Benjamini-Hochberg method was applied to obtain the false discovery rate for each analysis. Genes with a *q* value less than 0.05 and an absolute log_2_FC greater than 1.0 were considered differentially expressed.

#### Functional enrichment.

Gene Ontology biological process annotation enrichment using the biomaRt package (version 2.56.1) was performed in conjunction with AnnotationDbi (version 1.62.2). Active-subnetwork-oriented enrichment analyses were conducted using the PathfindR package (version 2.3.1), using lists of downregulated and upregulated genes from the differential expression analysis and using the Kyoto Encyclopedia of Genes and Genomes (KEGG) available within the PathfindR package. Furthermore, PathfindR facilitated the clustering of enriched pathways. This involved calculating pairwise κ statistics between the enriched terms and performing hierarchical clustering, with the optimal number of clusters automatically determined by maximizing of the average silhouette width. The clusterProfiler package (version 4.12.6) was implemented for gene cluster comparison.

### Statistics

Descriptive statistics, including the number of biological or technical replicates and the applied analysis, are reported in each figure legend. Experiments were performed at least twice. The normality of the samples was assessed using the Shapiro-Wilk test. Pairwise comparisons were conducted using a 2-tailed *t* test for independent samples, while multiple group means were evaluated using 1-way ANOVA with Tukey’s post hoc comparisons. The homoscedasticity of variance was examined using the Levene test. PCR data were analyzed using a 3 (time intervals) × 2 (mouse condition) full factorial ANOVA with the Greenhouse-Geisser correction. For all analyses, a *P* value less than 0.05 was considered significant. In the figures, the data represent mean ± SD, and significance is defined as *P* < 0.05. All analyses, except those related to RNA-Seq, were performed using GraphPad Prism version 9.0 (GraphPad Software).

### Study approval

The C57BL/6J and CD73-KO mice were housed in the Animal Facility of Facultad de Ciencias Químicas, Universidad Nacional de Córdoba registered with the NIH Office of Laboratory Animal Welfare (OLAW) under assurance no. F16-00193 (A5802-01) in strict accordance with the recommendations of the US Department of Health and Human Services *Guide for the Care and Use of Laboratory Animals* (National Academies Press, 2011). The studies were performed under the approval of the animal handling and experimental procedures of the Institutional Committee for the Care and Use of Laboratory Animals of Facultad de Ciencias Químicas, Universidad Nacional de Córdoba (permit CICUAL-Res:1746/2020).

The *Cd4^cre^*, *Hif1a^fl/fl^*, and *Vhl^fl/fl^* mice were housed at the Comparative Medicine Biomedicum and the Astrid Fagræus Laboratories, Karolinska Institutet, Stockholm, Sweden, according to directives and guidelines of the Swedish Board of Agriculture, the Swedish Animal Protection Agency, and the Karolinska Institutet (djurskyddslagen 1988:534; djurskyddsförordningen 1988:539; djurskyddsmyndigheten DFS 2004:4). The study was performed under the approval of the Stockholm North Ethical Committee on Animal Experiments (permit no. 1374-2020).

### Data availability

Values for all data points in graphs are reported in the Supporting Data Values file. The RNA-Seq dataset was obtained from the public repository GEO database (accession number GSE191081).

## Author contributions

GB designed the research study, conducted experiments, acquired and analyzed data, organized the database, performed the statistical analysis, and wrote the first draft of the manuscript. YLM, SDR, RL, and ZMCG contributed to the performing of the experiments. GB and SDR performed the RNA-Seq analysis. MPA conceived the project, designed and provided direction for the study, and corrected the manuscript. MR designed and supervised experiments. MR and SCR revised the final version of the manuscript and contributed to the interpretation of the results. All authors contributed to the article and approved the submitted version.

## Supplementary Material

Supplemental data

Supporting data values

## Version 1 07/01/2025

Electronic publication
