# Supplementary material for: Purinergic signaling modulates CD4+ T cells with cytotoxic potential during Trypanosoma cruzi infection
Source: J Clin Invest. 2025 Jul 1;135(13):e186785. doi: 10.1172/JCI186785 (PMC12208558; doi:10.1172/JCI186785)
Supplement: Supplemental data [file jci-135-186785-s124.pdf]

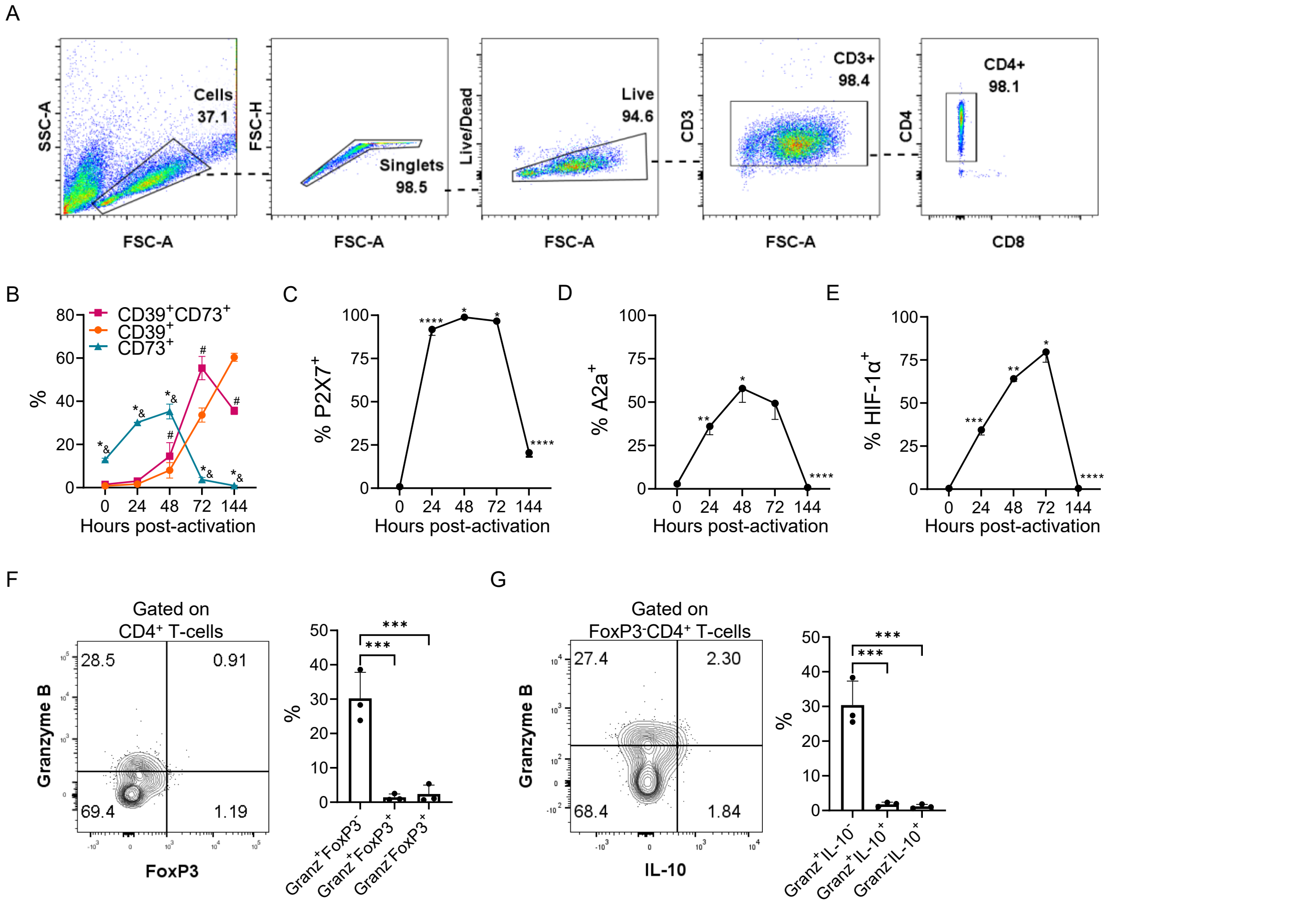

Supplemental Figure 1. In vitro activation of isolated CD4 T-cell. (A) Gating strategy employed to analyze splenic CD4⁺ T-cells by flow cytometry. **(B-E)** Frequency of CD39 and CD73 (B), P2X7R (C), A2aR (D), and HIF-1α (E) expression in CD4⁺ T-cells at indicated time point post-activation (n = 3/time). **(F-G)** Representative contour plots and frequencies of granzyme B and FoxP3 (F) or IL-10 (G) expression in total CD4⁺ T-cells or FoxP3⁻CD4⁺ T-cells at 72 hours post-stimulation (n = 3/group). Two-ways ANOVA followed by Tukey's post hoc test was conducted for panel **B** (*CD39⁺ vs CD73⁺, #CD39⁺ vs CD39⁺CD73⁺, &CD73⁺ vs CD39⁺CD73⁺; $p > 0.05$). Data in all panels were obtained from male mice. One-way ANOVA followed by Tukey's post hoc test was conducted for panels **C-G**. *$p < 0.05$, **$p < 0.01$, ***$p < 0.001$, ****$p < 0.0001$.

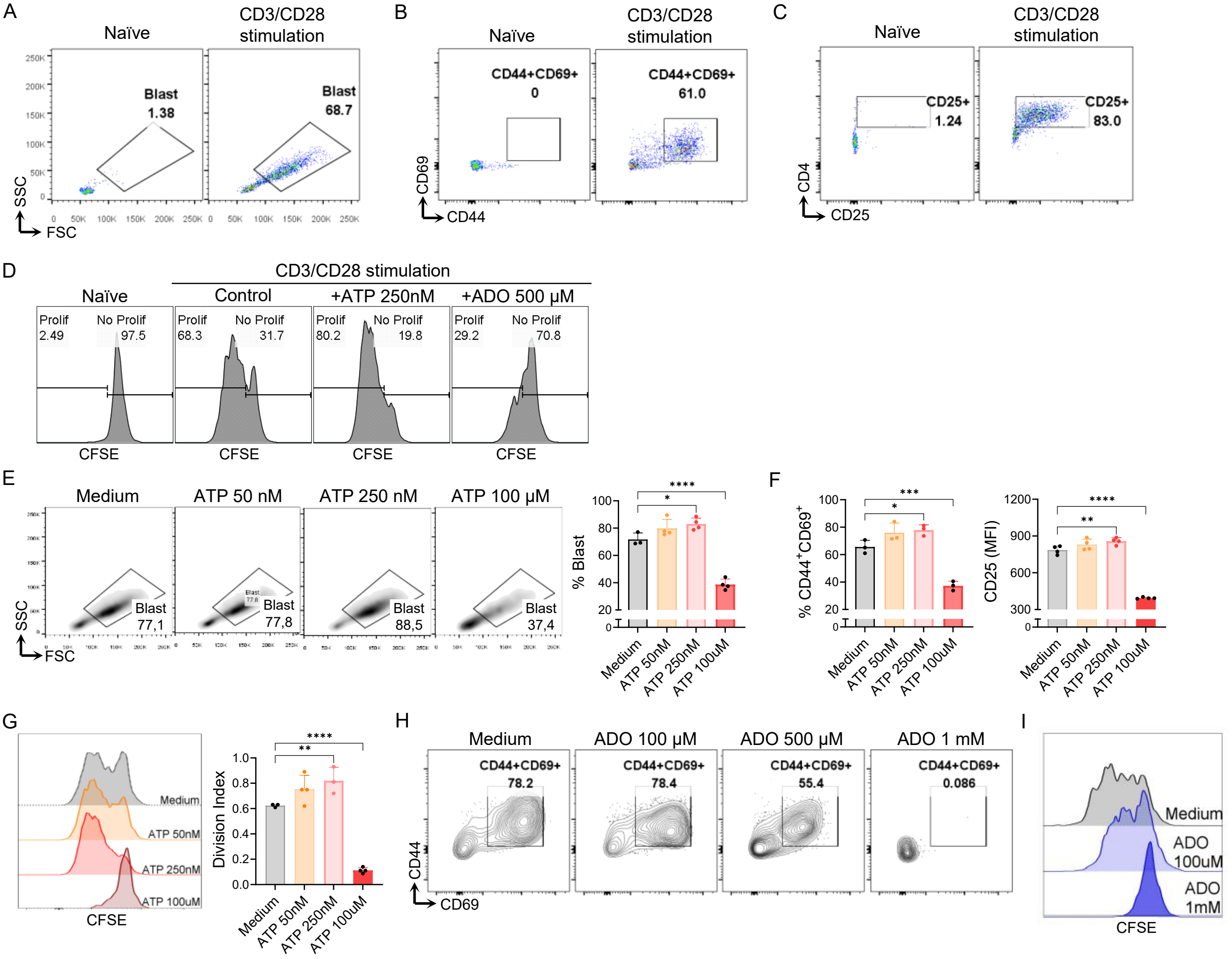

Supplemental Figure 2. **Concentration-dependent maturation effects of extracellular ATP on naïve T-cells. (A-D)** Dot plots showing the frequency of blasts (A), the expression of CD44 and CD69 (B), CD25 (C), and representative histograms of the proliferation rate (D) of naïve and 72h-activated $CD4^+$ T-cells. **(E-G)** Frequencies of blasts (E), $CD44^+CD69^+$, CD25 expression (F), and proliferation rate (G) in $CD4^+$ T-cells stimulated in the presence of ATP (50 nM, 250 nM, or 100 μM) or maintained in medium alone for 72 h (n = 4/group). **(H-I)** Representative CD44 and CD69 expression (H) and histograms of the proliferation (I) in $CD4^+$ T-cells stimulated in the presence of ADO (100 μM, 500 μM, or 1 mM) or maintained in medium alone for 72 h. Data in all panels were obtained from male mice. One-way ANOVA followed by Tukey´s post hoc test was conducted for panels **E-G**. Only the statistically significant differences versus medium are shown. $*p < 0.05$, $**p < 0.01$, $***p < 0.001$, $****p < 0.0001$.

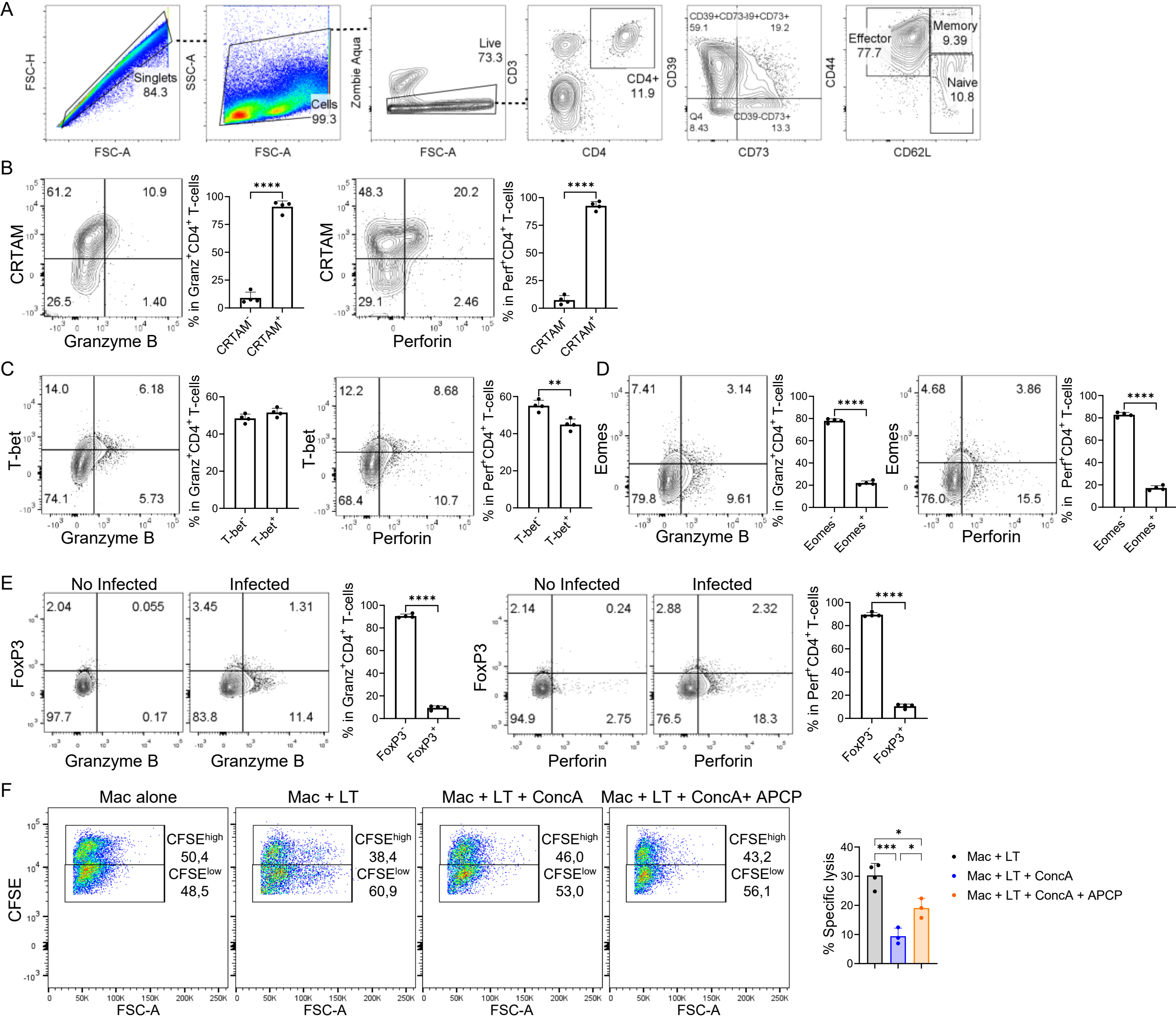

Supplemental Figure 3. Expression of transcription factors and cytotoxic capacity. (A) Representative gating strategy employed to determine CD39 and CD73 expression, alongside the frequencies of diverse subset cell populations within spleen CD4 T-cells. **(B-E)** Representative contours plots and frequencies of spleen cells expressing CRTAM (B), T-bet (C), Eomes (D), and FoxP3 (E) in Granz⁺ or Perf⁺CD4⁺ T-cells from 14dpi mice (n = 4/group). **(F)** Specific lysis of macrophages loaded with trypomastigote antigens following co-incubation with CD4 T-cells at a 1:8 ratio. CD4 T-cells obtained from infected mice (14dpi) were pre-treated with the perforin inhibitor concanamycin A (100 nM), either alone or in combination with APCP (100 μM), before co-culture with macrophages. One-way ANOVA followed by Tukey´s post hoc test was conducted for panels **B-F**.

Supplemental table 1. List and sequences of RT-PCR primers used.

Primer	Forward Sequence	Reverse Sequence
Hprt	CCCAGCGTCGTGATTAGC	GGAATAAACACTTTTTCCAAATCC
Ldha	TGGCAGACTTGGCTGACAG	ACCTTCACAACATCCGAGATTC
Vegfa	TAGAGTACATCTTCAAGCCG	TCTTTCTTTGGTCTGCATTC
P2rx7	AATCGGTGTGTTTCCTTTGG	CCGGGTGACTTTGTTTGTCT
Entpd1	AGCTGCCCCTTATGGAAGAT	TCAGTCCCACAGCAATCAAA
Cd73	AGAGTTCCTGCAAGTGGGTGGAATC	GACCACTCTGTTCCAGGGCTTTCG
A2AR	CCATCCGAATTCCACTCCGGT	TGACAGCACCCAGCAAATCGC
A2BR	GTGCTGGTGCTCACACAGAGC	TTTATACCTGAGCGGGACGCG

Supplemental table 2. List of reagents used.

Reagents	Concentration used	Company	Catalog Number
Adenosine (ADO)	500 µM	ThermoFisher	A10781
		Sigma-Aldrich	A4036
Adenosine 5′-triphospate (ATP)	250 nM / 100 µM	ThermoFisher	J61125
		Sigma-Aldrich	A6419
Adenosine 5′-(α,β-methylene) diphosphate (APCP)	100 µM	Sigma-Aldrich	M3763
Deferoxamine mesylate salt (DFO)	5 µM	Sigma-Aldrich	D9533
ZM-241385	1 µM	Sigma-Aldrich	Z0153
A-438079	25 µM	Sigma-Aldrich	A9736
CFSE	5 µM / 0,5 µM/ 2,5 µM	eBioscience	65-0850
Recombinant Murine IL-2	20 ng/mL	PeproTech	212-12-50UG
Rat IgG1 kappa Isotype Control	40 µg/mL	eBioscience	14-4301-82
Mouse IL-6 Recombinant Protein	10 pg/mL	PeproTech	216-16
IL-6 Monoclonal Antibody (MP5-20F3)	5 ug/mL	eBioscience	16-7061-81
anti-mouse IFNγ (XMG1.2)	40 µg/mL	BioXCell	BE0055
Concanamycin A	100 nM	Sigma-Aldrich	C9705

Supplemental table 3. List of antibodies used.

Abs to Flow Cytometry	Clone	Company	Catalog Number
Live/Dead		Invitrogen	L34968
		BioLegend	423102
CD45	30-F11	BioLegend	103114
CD3e	17A2	eBioscience	48-0032-82
	145-2C11	BD	553063
CD4	GK1.5	BD	563331
		BioLegend	100410
CD44	OX-49	eBioscience	67-0441-82
	IM7	BioLegend	103007
CD69	FN50	eBioscience	47-0691-82
CD25	PC61	BD	561257
CD62L	MEL-14	BioLegend	104411
CD39	24DMS1	eBioscience	46-0391-82
CD73	eBioTY/11.8 (TY/11.8)	eBioscience	48-0731-82
		Biolegend	127215
P2X7	1F11	BioLegend	148708
A2a	Polyclonal	Invitrogen	PA1-042
HIF-1α	EP1215Y	Abcam	ab51608
	H1alpha67	Novus Biologicals	NB100-105
Ki67	16A8	BioLegend	652422
Granzyme B	GB11	BioLegend	515403
	QA18A28	BioLegend	396410
Perforin	S16009A	BioLegend	154305
IFN-γ	XMG1.2	BioLegend	505836
anti-rabbit IgG	Poly4064	BioLegend	406414
CD11b	M1/70	eBioscience	11011282
Eomes	X4-83	BD	567168
T-bet	4B10	BioLegend	127215
CRTAM (CD355)	11-5/CRTAM	BioLegend	142014
FoxP3	FJK-16s	Invitrogen	45-5773-82
IL-10	JES5-16E3	eBioscience	17-7101
